# Occupational heat stress, heat-related effects and the related social and economic loss: a scoping literature review

**DOI:** 10.3389/fpubh.2023.1173553

**Published:** 2023-08-02

**Authors:** Manuela De Sario, Francesca Katherine de'Donato, Michela Bonafede, Alessandro Marinaccio, Miriam Levi, Filippo Ariani, Marco Morabito, Paola Michelozzi

**Affiliations:** ^1^Department of Epidemiology Lazio Regional Health Service, Rome, Italy; ^2^Occupational and Environmental Medicine, Epidemiology and Hygiene Department, Italian Workers' Compensation Authority (INAIL), Rome, Italy; ^3^Epidemiology Unit, Department of Prevention, Central Tuscany Local Health Authority, Florence, Italy; ^4^Regional Centre for the Analysis of Data on Occupational and Work-Related Injuries and Diseases, Central Tuscany Local Health Authority, Florence, Italy; ^5^Institute of Bioeconomy, National Research Council (IBE-CNR), Florence, Italy

**Keywords:** productivity loss, workers, climate change, occupational heat exposure, economic costs, scoping review

## Abstract

**Introduction:**

While there is consistent evidence on the effects of heat on workers' health and safety, the evidence on the resulting social and economic impacts is still limited. A scoping literature review was carried out to update the knowledge about social and economic impacts related to workplace heat exposure.

**Methods:**

The literature search was conducted in two bibliographic databases (Web of Science and PubMed), to select publications from 2010 to April 2022.

**Results:**

A total of 89 studies were included in the qualitative synthesis (32 field studies, 8 studies estimating healthcare-related costs, and 49 economic studies). Overall, consistent evidence of the socioeconomic impacts of heat exposure in the workplace emerges. Actual productivity losses at the global level are nearly 10% and are expected to increase up to 30–40% under the worst climate change scenario by the end of the century. Vulnerable regions are mainly low-latitude and low- and middle-income countries with a greater proportion of outdoor workers but include also areas from developed countries such as southern Europe. The most affected sectors are agriculture and construction. There is limited evidence regarding the role of cooling measures and changes in the work/rest schedule in mitigating heat-related productivity loss.

**Conclusion:**

The available evidence highlights the need for strengthening prevention efforts to enhance workers' awareness and resilience toward occupational heat exposure, particularly in low- and middle-income countries but also in some areas of developed countries where an increase in frequency and intensity of heat waves is expected under future climate change scenarios.

## 1. Introduction

There is a consistent body of evidence that high outdoor and indoor temperatures have adverse health effects in exposed workers (1–4). Workers are normally healthier than the general population, but they, especially those severely exposed and engaged in heavy workloads, may be equally affected by heat stress when the thermoregulatory capacity of the body is disrupted, and physiological pathways resulting in heat-related illness, acute outcomes (e.g., myocardial infarction), or exacerbations of pre-existing diseases (e.g., cardiovascular and respiratory outcomes) are activated (2). Individuals working in the heat are also prone to physical strength losses and cognitive function impairments, leading to work-related injuries (3), missed workdays, and productivity reductions (4) and, in the long term, may develop chronic kidney impairment (5, 6). Health and productivity outcomes related to heat strain have a huge impact in terms of social and economic costs on the different actors involved (7): the workers themselves due to the temporary or permanent health and quality of life impairments and missed wages, the farm or factory due to necessity of maintaining production despite employees absences or output reductions, the healthcare system due to the healthcare expenditures due to workers seeking care, the social security or insurance system due to reimbursements to laborers for injuries, permanent disability, or occupational diseases, and the whole country or region in terms of reductions of the gross domestic product due to production losses in specific economic sectors. Moreover, climate change is expected to worsen heat exposure in some regions exceeding work-related productivity thresholds (8).

Heat exposure in the workplace is a growing hazard throughout the world, considering climate change scenarios showing a universal increase in heat extremes virtually in every region, but larger in Central and South America, the Mediterranean region, north Africa, the Arabian Peninsula, India, and Southeast Asia (9). Most of the affected regions are low-income economies mostly relying on manual labor and manufacturing work with agriculture and construction being the economic sectors at higher risk of heat exposure and at higher workload intensity than others. The quantification of economic impacts of heat exposure in the workplace is of worth for individual companies, labor policymakers, insurance companies, but also for occupational safety and healthcare systems and should be taken into account when analyzing markets and economies at both the local and global scale. The knowledge of economic losses related to heat may serve as a basis to plan prevention measures at company level with a view on cost–benefit analysis, to set up specific heat adaptation policies, or to strengthen social security systems by enclosing climate risk concerns, especially toward poorer population and countries (10).

Differently from the strong evidence available on the effects of heat on workers' health and safety, there is still limited but growing evidence on the resulting social (7, 11) and economic impacts (12). The lack of standardized methodologies for evaluation (epidemiological vs. econometric studies), as well as in the operational definitions of productivity loss (lost worktime, reported physical and cognitive performance reductions, and work output reductions in case of manual workers), heat-related productivity losses, and economic costs (i.e., lost salaries and wages due to fatigue/sickness, cost per compensable claim, and healthcare costs related to treatment and rehabilitation) make it difficult to have consistent findings and clear trends. Despite the close connection between indicators of social and economic heat-related impacts and the common underlying causal pathways, no previous literature review considered both social and economic losses related to heat at the same time.

As part of the Italian WORKLIMATE Project (https://www.worklimate.it) funded by the Italian Workers' Compensation Authority (INAIL), a literature review was carried out to update the evidence on both social and economic impacts related to workplace heat exposure. To address such a comprehensive research question, a scoping review was considered to be more suitable, as suggested also by other authors (13), to address the whole body of evidence deriving from different type of studies (i.e., epidemiological and economic modeling studies).

## 2. Methods

The scoping literature search was conducted in two bibliographic databases (Web of Science and PubMed), using both free terms and controlled vocabulary (Supplementary Table 1) to select studies published since 2010 to April 2022. Since previous reviews considered impacts of occupational heat stress on workers' productivity (7, 11, 14) and on economic losses (12) separately, and in consideration of the interconnections between work performance, workers' health and safety, and monetary costs, the outcomes of interest were both social impacts related to workers (i.e., work hours losses and work absences), and economic impacts for a specific group of workers or economic sector (i.e., monetary costs associated to production losses) or for social security systems (i.e., compensation for work-related injuries and diseases). Both indoor and outdoor occupational heat exposure and all potentially exposed economic sectors and tasks (e.g., manual workers) were considered. The first group of relevant studies were from epidemiological studies (both qualitative and quantitative) on workers estimating productivity losses in the field or estimating costs related to occupational heat-related illnesses and injuries. The second category of suitable studies was represented by recently conducted economic studies adopting several approaches such as structural economic models and econometric models, to estimate the impacts of climate change on labor productivity and related economic costs, using occupational health and safety recommendations in an entire economic sector and for regional or global economies. Experimental studies (e.g., on physiological responses), epidemiological studies on occupational heat-related illnesses not estimating economic implications, studies focusing only on the impact of heat exposure on workers' cognitive functions, and studies on other occupational exposures (e.g., cold, air pollution) were excluded. Only original studies were retrieved, while literature reviews (7, 11, 12, 14–16) were excluded but used to screen for additional relevant studies, as well as the 6th assessment report of the Intergovernmental Panel on Climate Change (https://www.ipcc.ch/report/ar6/wg2/) (8). The selection of studies and data extraction were conducted according to PRISMA guidelines (17). The outcomes considered were as follows:

- Lost productivity estimated or perceived by the worker associated with the heat exposure.
- Economic costs associated with heat-related injuries or hospitalizations in workers.
- Projections of productivity losses due to heat and related economic costs for current climate or under climate change scenarios.

Given the heterogeneity of study designs, methods for estimating costs or productivity, outcomes considered, and occupational sectors investigated, a narrative synthesis was undertaken by grouping studies by design (epidemiological vs. economic studies).

## 3. Results

**Figure 1:**
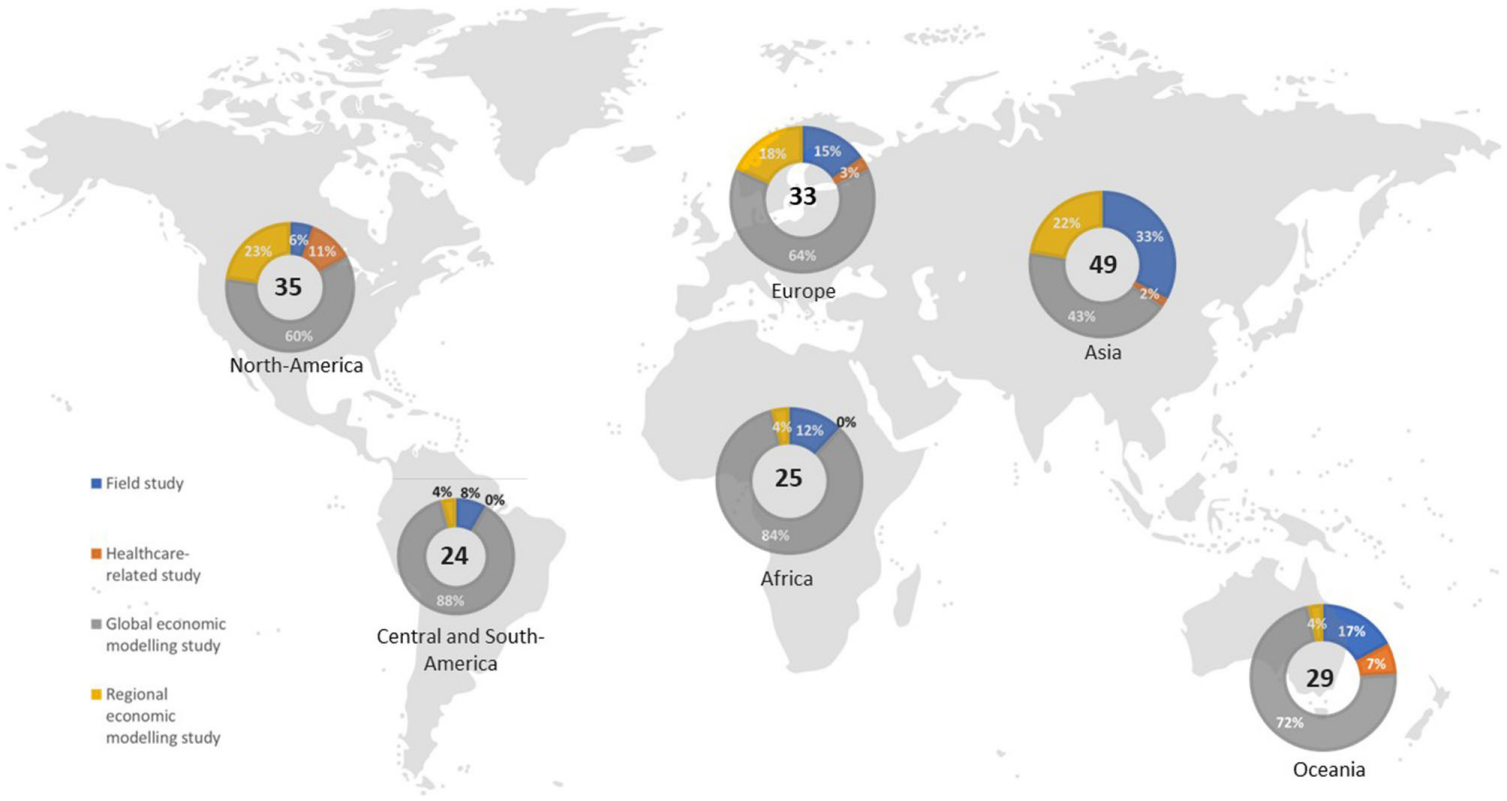
Geographical distribution of included studies based on the study design by continents.

A total of 8,151 potentially relevant records were identified after duplicates were removed, of which 104 were identified from previous reviews on the topic (7, 11, 12, 14–16) or other sources (Supplementary Figure 1). Out of these, 137 were assessed as full texts because potentially relevant, and, finally, 89 studies were included in the qualitative synthesis. The largest number of studies was carried out in Asia (49 studies) and the lowest in Central and South America (24 studies) (all totals include the 21 global economic modeling studies) (Figure 1). Field studies accounted for a larger proportion of studies in Asia, Oceania, and Europe, healthcare-related studies were more prevalent in North America and Oceania, and regional modeling studies were mostly conducted in North America, Europe, and Asia.

Table 1 describes the results of the epidemiological studies (*n* = 40), including 32 field studies and 8 studies estimating healthcare-related costs.

**Table 1:** Results of epidemiological studies estimating productivity, social, or economic losses related to occupational heat exposure.

**Reference**	**Study type**	**Country**	**Heat exposure**	**Study population**	**Study period and duration**	**Productivity or cost calculation (unit measure)**	**Heat-related economic loss analysis**	**Results**
**Field studies**								
Amini et al. (18)	Field study	Southwest Iran	Predicted mean vote (PMV) index	Agriculture workers	2016	Manpower productivity index at area level	Productivity loss due to heat calculated based on equation in Mohamed (2005) at area level	A strong and significant (P < 0.05) relationship between temperature index in the cold regions was found. In the hot regions, all three main environmental variables have a strong and significant correlation (P < 0.05) with the P index.
Bonafede et al. (19)	Field study	Italy	Perceived stress from heat and heat waves	345 workers in several occupational sectors indoor (with and without air conditioning) and outdoor	June–October 2022	na	Perceived labor productivity loss due to heat	73% of workers perceived heat completely or very much as an important contributor to productivity loss (mean score of 3.93 on a scale of 1 to 5).
Budhathoki and Zander (20)	Field study	Nepal	Perceived stress from heat and heat waves	350 farmers	2012–2017	na	Perceived labor productivity loss due to heat	Farmers' perceived heat stress levels, and the number of associated illnesses or symptoms, significantly increase labor productivity loss during heat waves (p < 0.05). Residency in urban areas, access to weather information, past implementation of prevention measure increases labor productivity losses perception due to heat.
Dally et al. (21)	Field study	Guatemala	Wet-bulb Globe Temperature (WBGT)	4, 095 male sugarcane cutters	November 2015 to May 2016 harvest season.	Daily output (average daily tons)	Change in productivity due to heat (lag 0–5) estimated by distributed lag non-linear models	The cumulative effect on tons of sugarcane cut for workers with impaired kidney function who experienced exposure to a WBGT of 34°C is estimated to be a loss of 1.16 (95% confidence interval (CI): −2.87, 0.54) tons over the next five days compared to if they were exposed to a WBGT of 29°C. The estimated cumulative effect on tons of sugarcane cut by workers with functioning kidneys was 0.59 tons (95% CI: −2.05, 0.87) less.
Das (22)	Field study	India (two cities)	Heat wave days	150 low-income urban informal workers (mostly outdoor)	April–May 2013	Worktime (in hours)	Change in time allocation and worktime loss during heat wave compared to normal days (adjusting for workplace, family size and income, and worker's health)	The results show that workers work 1.19 h less and spend 0.46 h less at home, and they rest 1.65 h longer on average on a heat wave day than on a normal summer day. Worktime loss is more for people doing manual work and having health problems
Davey et al. (23)	Field study	UK	Perceived heat stress and heat-related illness	Healthcare workers	May and August 2020	na	Perceived cognitive and physical performance reductions due to heat stress and PPE	Heat stress impaired both cognitive and physical performance of workers. Respondents reported that PPE impaired their physical performance at work (76%) and made their job more difficult (92%).
Cortez (24)	Field study	Nicaragua	Wet-bulb Globe Temperature (WBGT)	22 sugarcane workers	2006/2007 harvesting season (15 days)	Work output (in daily tons)	Change in production output related to water intake (no analysis of production output and temperature)	Output production increased significantly among those best hydrated, from 5.5 to 8 tons of cut sugarcane per worker per day.
Gun and Budd (25)	Field study	Australia	Wet-bulb Globe Temperature (WBGT)	43 male sheep shearers	January–March of two consecutive years (54 days)	Work output: hourly number of sheep shorn and wool bales pressed recorded in a tally book	Change in productivity due to heat estimated by linear regression analysis between productivity and thermal stress variables	Shearers under thermal discomfort tended to be less productive (*r* = −0.32, *b* = −3.0, *p* = 0.04). No influence by age, dehydration, alcohol consumption, and obesity on productivity loss.
Lamb and Kwok (26)	Field study (longitudinal within-subjects design)	New Zealand	Indoor Environmental Quality (thermal stress including both cold and heat stress)	114 office workers	8 months	Several measures of productivity: 11-point scale of work performance relative to average perceived performance; 11-point scale of level of distraction from work; Reaction time and accuracy measured by Stroop test; 9-point scale of tiredness; 11-point scale of motivation.	Work performance reduction related to thermal discomfort (both heat and cold) evaluated descriptively. Change in productivity related to a combined exposure index (noise, temperature, light) also evaluated.	Thermal comfort did not significantly affect work performance.
Langkulsen et al. (27)	Field study	Thailand	Wet-bulb Globe Temperature (WBGT)	21 workers in pottery industry, power plant, knife industry, construction site, and agricultural site	October 5 to October 16, 2009	Work output	Productivity loss measured as percent change of the daily work output due to heat relative to the daily work output	In knife and agriculture workers no losses of productivity. In power plant workers not applicable. In pottery and construction workers losses of productivity from 10 to 60%.
Lao et al. (28)	Field study (qualitative study)	South Australia	na	32 male outdoor workers	July 2014	na	Heat impact on productivity evaluated in a narrative way by workers in focus group	Narratives revealed that working on hot days could affect health and wellbeing, and work productivity
Lee et al. (29)	Field study	India and Singapore	Perceived heat stress	165 hospital workers using PPE during COVID-19 epidemic	May-June 2020	na	Perceived productivity loss due to heat stress and PPE self-assessed from questionnaire	Workers reported a reduced productivity due to heat and when wearing PPE
Li et al. (30)	Field study	China	Wet-bulb Globe Temperature (WBGT)	16 rebar workers	summer 2014	Worktime: time for labor productivity of direct worktime, indirect worktime, and idle time measured by observers	WBGT-productivity relationship evaluated in regression models (adjusted for age, BMI, work experience)	High-temperature environments decrease labor productivity, with the percentage of direct worktime decreasing by 0.57% and the percentage of idle time increasing by 0.74% when the WBGT increased by 1 °C.
Lundgren et al. (31)	Field study	Chennai, India	Wet-bulb Globe Temperature (WBGT)	77 workers in industrial, service, and agricultural sectors (most workers with moderate to heavy work)	January–February and April–May	na	Productivity loss estimation based on Predicted Heat Strain (PHS) model from core temperature and maximum water loss as a function of ISO standard guidelines.	Heat strain was related to productivity loss in the PHS model in all workplaces, apart from the laundry facility, especially during the hot season
Lundgren-Kownacki et al. (32)	Field study	India	Perceived heat stress	87 migrant brick kiln workers in summer and 61 in winter	June–July 2013, March–April 2014 (hot season); February 2013, January–February 2015 (cool season)	na	Perceived productivity loss due to heat: absenteeism/taken sick leave due to heat; Less productivity/more time to complete task/work extra hours; Irritation/interpersonal issues; Wages lost	16% of workers in summer reported absenteeism/sick leave due to heat stress, 48% reported less productivity
Mathee et al. (33) - HOTHAPS study	Field study (qualitative study)	South Africa	Perceived heat stress	151 workers involved in sun-exposed occupations	March 2009	na	Perceived productivity loss due to heat. No analysis was carried out, only narrative description of interviews.	Where daily maximum temperatures may reach 40°C, workers reported a wide range of heat-related effects, leading to difficulty in maintaining work levels and output during very hot weather
Messeri et al. (34) (EU HEAT-SHIELD project)	Field study	Italy	Perceived heat stress	104 native and migrant workers in agriculture and construction	Summer months of 2017	na	Perceived productivity loss due to heat (the worker noticed to be less productive during a heat wave or need more energy for the same work)	Migrant workers declared that work required greater effort than do native Italian workers (Chi squared p = 0.001) but reported less impact from heat on productivity (Chi squared p = 0.014) and on thermal discomfort.
Messeri et al. (35) (WORKLIMATE project)	Field study	Italy (mostly areas from Center-South of Italy)	Perceived heat stress	191 hospital workers using PPE during COVID-19 epidemic	June–October 2020	na	Perceived productivity loss due to heat and PPE	A great number of HCW (81%) self-reported a productivity loss related to heat stress exposure. The productivity loss was significantly correlated (*p* < 0.001) to the perception of thermal sensation due to the use of PPE.
Morabito et al. (36) (HEAT-SHIELD)	Field study	Florence and Guangzhou	Wet-bulb Globe Temperature (WBGT)	18 outdoor workers in agriculture	Summer 2017–2018	na	Productivity loss (% of reduced work capacity) in outdoor workers for moderate (300 W) work activities in sun and shady areas assessed by risk functions based on ISO standard and on epidemiological data (37). Economic costs (euros) estimated from workers' salaries multiplied for productivity losses.	The hourly economic cost in Italian farm related to the productivity loss in the sun during the typical working time ranged between €5.7 and €8.0, higher than productivity loss in the shade. The productivity loss values estimated in the sun in Guangzhou were 7.3, 8.2, and 8.3 times higher than the values estimated in Florence and even greater considering shade conditions.
Nunfam et al. (37)	Field study	Ghana	Perceived heat stress	320 miners	October 2017–January 2018	na	Causal analysis evaluating the relationship between heat exposure and productivity outcomes by structural equation models. Evaluation of moderation effect by adaptation strategies and demographic and work variables.	Heat exposure had a significant direct effect on the productivity outcomes of mining worker. This effect was moderated by barriers to adaptation strategies, mediated through adaptation strategies (not significantly), and controlled by some demographic and work-related variables.
Pradhan et al. (38) (HOTHAPS study)	Field study	Nepal	Wet-bulb Globe Temperature (WBGT) and Humidex	120 workers indoor and outdoor	2010	Worktime: Average work hours by season (work efficiency)	Descriptive comparison of worktime across months.	Duration of work is longer in summer due to longer days and more frequent rests or longer mid-day off.
Quiller et al. (39)	Field study	Washington, US	Wet-bulb Globe Temperature (WBGT)	46 tree harvesters	2015 August and September	Work output: Total weight of fruit bins collected per time worked (kg/hours)	WBGT-productivity relationship estimated by linear mixed effects models (adjusted models for work experience, gender, price paid per bin, BMI, and shift duration)	There was a trend of decreasing productivity with increasing WBGT, but this was not statistically significant (significant only in unadjusted model).
Sadiq et al. (40)	Field study	Nigeria	Wet-bulb Globe Temperature (WBGT)	396 maize farmers	July to September, 2016	Work output: number of ridges cultivated during the working hours	WBGT-productivity relationship estimated by multiple linear regression adjusting for body mass index (BMI), age, and gender.	Productivity was significantly higher between the hours of 6–9 am (p < 0.001) and 12–3 pm (p < 0.001), compared to the hours of 9 am−12pm (p < 0.001)- For temperature increases, productivity decreases (beta coefficient = −0.6, *p*-value < 0.001).
Sahu et al. (41)	Field study	India	Wet-bulb Globe Temperature (WBGT)	124 rice harvesters	April-June 2011	Work output measured in terms of volume or quantity of items collected (rice bundle)	Productivity loss estimated for WBGT exceeding the standard (26–32°C) corresponding to 30–38°C of air temperature.	High heat exposure in agriculture caused heat strain and reduced work productivity (−5% per 1°C). This reduction will be exacerbated by climate change and may undermine the local economy
Sett and Sahu (42)	Field study (longitudinal study)	West Bengal, India	Wet-bulb Globe Temperature (WBGT)	120 female brickfield	October 2008 to May 2009 (first session), from October 2009 to May 2010 (second session), and then from October 2010 to May 2011 (third session)	Work output (number of bricks molded or carried per person per week) recorded on a weekly basis from the record register book	Productivity loss estimated for WBGT exceeding the standard (26–32°C) corresponding to 30–38°C of air temperature. Comparison of wages by season. Comparison of walking speed by season.	Productivity loss for every degree rise in temperature was about 2%. Wages of the female workers vary from 800 INR/week in the extreme summer months to 1,500 INR/week in the winter months. Reduced walking speed in summer compared to winter.
Singh et al. (43)	Field study (qualitative study)	Australia	n.a.	47 workers outdoor in several industries (encl. Farming, construction)	Summer 2010	na	Perceived productivity loss due to heat. No analysis was carried out, only narrative description of interviews	All interviewees reported that excessive heat exposure presents a significant challenge for their industry or activity. People working in physically demanding jobs in temperatures>35°C frequently develop symptoms, and working beyond heat tolerance is common. To avoid potentially dangerous health impacts, they must either slow down or change their work habits. Such health-preserving actions result in lost work capacity.
Vanos et al. (44)	Field study	Ontario, Canada	Wet-bulb Globe Temperature (WBGT)	Outdoor laborers at an industrial worksite	2012–2018 (May–October)	na	Worktime loss due to heat estimated by risk functions for different WBGT levels. Loss of money (Canadian dollars) due to heat per 15-min work interval estimated by laborer type (*via* hourly wages).	On average, 22 h per worker were lost each summer (ca 1% of annual work hours) as a result of taking breaks or stopping due to heat. This amount of time corresponded to an average individual loss of 1100 Canadian dollars to workers or the company.
Venugopal et al. (45)	Field study	South India	Wet-bulb Globe Temperature (WBGT)	84 steel workers	April 2014	na	Perceived productivity loss due to heat stress defined as: loss in production, not achieving work targets, loss of workdays/work hours due to fatigue/exhaustion, sickness/hospitalization, and/or wages lost due to heat or heat-related illnesses	Workers exposed directly to heat sources reported higher productivity losses than other workers. Heat exposure was related to greater absenteeism (+1% increase), less productivity (−10.6%), larger work extra hours (26.9%), and increase in irritation/interpersonal issues (+7.7%)
Venugopal et al. (46)	Field study	India	Wet-bulb Globe Temperature (WBGT)	Several occupation types (indoor and outdoor, heavy, moderate, and light)	Cooler (2012) and hotter (2013) seasons	na	Perceived productivity loss due to heat stress defined as: loss in production, or not achieving set work targets, or loss workdays/work hours due to fatigue/exhaustion, or sickness/hospitalization, and/or wages lost due to heat or heat-related illnesses.	Of the 442 workers, approximately 62% reported reduced productivity by not achieving targets, 30% reported absenteeism as a reason for productivity loss and 25% of workers reported lost wages due to fatigue/sickness due to workplace heat stress. Males and workers with heavy workload (especially outdoor workers) were significantly affected by heat-related productivity losses.
Yi and Chan (47)	Field study	Hong Kong	Wet-bulb Globe Temperature (WBGT)	14 male construction workers	August and September 2016	Productivity measures as direct worktime (Make use of wrenches to connect, cut, band, and modify reinforcing steel bars, Place reinforcing steel bars, Modify reinforcing steel bars, Carry reinforcing steel bars, Use meter sticks for measurements, Bending)	WBGT-productivity relationship estimated by linear regression models (adjusted for age, work duration, cigarette intake, alcohol drinking consumption, weight, and work intensity)	Heat stress reduces construction labor productivity, with the percentage of direct worktime decreasing by 0.33% when the WBGT increased by 1 °C.
Zander et al. (48)	Field study	Australia	Self-reported heat stress	1726 workers in several occupation types (both outdoor and indoor)	2013/2014	Productivity measured as absenteeism, or presenteeism (less productive days) from the work productivity and activity impairment (WPAI) questionnaire	Self-reported estimates of absenteeism and reductions in work performance (presenteeism) caused by heat. Total production loss (TPL) was calculated as sum of PLA is the annual production loss from absenteeism and PLP the annual production loss from presenteeism. PLA was calculated for each individual as NA x DI where NA=number of days absent per year due to heat stress and DI=daily income. PLP was calculated for each individual as HL x NP x HI, where HL=hr lost per less productive day, NP=number of days per year of lower productivity, and HI=hourly income. HL was calculated as p x H where p=the percentage by which productivity was reduced on less productive days and H=number of hours per day spent working for payment. Annual total productivity loss is adjusted for compensation and in US$.	The individual annual economic losses due to heat were US$655 per person, which translates to an economic burden totaling US$6.2 billion in Australia. Percent reduction in productivity 30% in both males and females. Annual total productivity loss higher for workers with medium-high proportion of time outdoor and higher physical exertion, higher for machinery operators/drivers. Across the whole sample, of whom 70% were less productive and 7% absent on at least one day per year owing to heat, the total economic loss was US$711 per person per year, which was reduced to US$655 if compensatory behavior is accounted for (i.e., compensate for productive time lost by working longer hours). This was, on average, 1.2% of respondents' gross annual income.
Zander and Mathew (49)	Field study	Urban Malaysia	Self-reported heat stress	514 workers several occupation types (both outdoor and indoor)	2017–2018	Productivity measured as absenteeism, or presenteeism (less productive days) from questionnaire	Self-reported estimates of work absenteeism and reductions in work performance caused by heat. Individual economic losses estimated from productivity loss per daily average income per number of affected days.	The median number of days in a year on which people felt their productivity had been compromised because of heat stress was 29. On those days, half of the respondents felt their work capacity had been at least halved. The estimated median annual loss from reduced productivity was 257 €, nearly 10% of respondents' median annual income. Annual losses greater in medium-heavy physical exertion categories and heavy mental exertion categories.
**Studies evaluating healthcare-related costs**								
Bonauto et al. (50)	Descriptive study of compensation claim data related to heat	US Washington State	n.a.	All work sectors (480 compensation claims for heat-related illness in the study period)	1995–2005	Both compensable and non-compensable claims were included. Non-compensable claims (medical-only). Compensable claims involve 4+ lost workdays, a permanent partial disability award, were kept on-salary by the employer or resulted in a fatality. Workers' compensation claim costs represent those paid to date for closed claims. For open claims on the date of extraction, the claim costs represent those paid to date and an estimate by the L&I workers' compensation case reserve unit of future expected claim costs. Indirect costs to employers and workers and the administrative costs of managing the claim are not included in the claim costs.	Descriptive analysis of heat-related illness compensation claims and risk factors (outdoor/indoor, comorbidity, hours of the day, acclimatization) and related costs.	Median cost for all compensable and not compensable claims for heat-related illness was 537 USD (mean 1,864 USD), higher than for total claims (not only for heat-related injuries). Also median cost for non-compensable claim was higher for heat-related illness than for total claims (513 vs. 251 USD). Median cost per compensable claim for heat-related illness was 1,916 USD, lower than for total claims (4,771 USD). For time loss claims, the median number of working days lost was 6 (46 days on average).
**Studies evaluating healthcare-related costs**								
Fortune et al. (51)	Descriptive study of heat-related injuries	Ontario, Canada	na	All work sectors (612 compensation claims for heat-related illness in the study period)	2004–2010	na	Lost time claims related to excess heat exposure. Incidence rates calculated using denominator estimates from national labor market surveys and estimates were adjusted for workers' compensation insurance coverage. Proportional morbidity ratios were estimated for industry, occupation and tenure of employment.	Incidence of heat illness and lost time claims related to excess heat exposure highest in the June to August period. A total of 40% of all heat illnesses were clustered in epidemics over contiguous days. The rates of lost time claims were highest among workers aged 15–24, males, and among manufacturing (25%), Government Service (15%), construction (10%), and self-insured public sector employers (10%) sectors.
Hesketh et al. (52)	Descriptive study of heat-related injuries	US Washington State	Maximum daily and 3-day temperature (°F) > 89°F (threshold to protect workers)	645 heat-related injuries occurred in all work sectors	2006–2017	Worktime loss due to heat-related injuries. Claim costs (in USD) for compensable and non-compensable (medical aid only) claims, excluding indirect costs to employers and workers and the administrative costs of managing the claim.	Descriptive analysis of time losses and costs per injury	For time loss claims, median time loss of 13 work days related to heat injury (mean of 93 days). Higher median costs for heat-related injuries than for total injuries for both all claims (909 USD and 800 USD, respectively) and non-compensable claims (876.9 vs. 560 USD). For compensable claims higher costs for total than for heat-related injuries.
Ma et al. (53)	Time-series study of the relationship between temperature and occupational injuries	China	Wet-bulb Globe Temperature (WBGT)	all work sectors	2011–2012	The daily insurance payouts calculated by aggregating amounts of individual payouts and also showed as USD	Time-series study (Distributed Lag non-linear models) to examine the association between heat stress (WBGT values) and work-related injuries and insurance payouts. To calculate the measures of injury claims and the cost of compensation attributable to high WBGT values, the daily maximum WBGT and the corresponding relative risks were combined to assess the attributable numbers of each day.	4.1% of insurance payouts was attributable to heat stress (all days in the study period with WBGT>25°C), corresponding to 11.58 million Chinese Yuan. Stronger risk of heat-related injuries in workers aged 35–44 and < 35, workers employed in small enterprises, and workers with intermediate education level, and in workers severely injured. Significantly higher costs in males (but significant impact also in females), medium-sized enterprises, workers with intermediate education level.
Martínez-Solanas et al. (54)	Time-series study of the relationship between temperature and occupational injuries	Spain	Extreme cold and heat defined as temperatures below the 2.5th and above the 97.5th percentiles, and moderate heat and cold between minimum mortality temperature and the extreme threshold, respectively	Occupational injuries in specific economic sectors (no information about indoor and outdoor)	1994–2013 (both heat and cold)	Occupational injuries that caused at least one day of leave were considered. Costs (euros) estimated based on a previous study on the costs of occupational injuries in the Catalonia region (55) estimating (a) costs associated with maintaining production (including overtime payments and costs of replacement and training), (b) long-term lost incomes (total income lost when a worker suffers an injury and cannot come back to work), (c) health costs associated with costs of treatment and rehabilitation, and (d) costs of pain and suffering (level of disability).	Time-series study (Distributed Lag non-linear models) between daily maximum temperature and the daily count of occupational injuries. Analyses of economic losses due to working at extreme temperatures (total economic costs due to non-optimal temperature per year) by multiplying cost of each lost workday due to injury for the number of working days lost due to non-optimal temperatures by considering the empirical distribution of the number of days of sick leave for each category of leave duration and the attributable number for both cold and heat.	0.67 million (95%CI: 0.60–0.73) person-days of work lost every year due to temperature. 319.39 million euros annually related to heat (297.82 for moderate heat, 21.57 for extreme heat). Annual costs related to moderate and extreme heat from pain and suffering: 182.97 million euros, maintaining production: 59.21 million euros, long-term lost incomes: 49.16 million euros, and health costs: 28.06 million euros.
Rameezdeen and Elmualim (56)	Descriptive study of heat-related injuries	Adelaide, Australia	Heat wave: five or more consecutive days of maximum temperature in excess of 35°C or three or more consecutive days of temperature in excess of 40°C	Construction sector (29,438 compensation claims during the study period)	heat waves 2000–2010	Compensation claims and costs (Australian dollars)	Descriptive analysis of occurrence and severity of construction accidents by worker's characteristics. Compensation claims recorded during the heat wave periods were compared with those during similar “control periods”.	The average cost per compensation claim was 26,381 Australian dollars during heat waves compared to 12,747 Australian dollars during control periods. Worker characteristics (older workers 55+ years old, new workers, male workers), type of work (civil subsector, slight over-representation of bricklayer, carpenter, electrician, mechanic, and plant operator), work environment, and agency of accident increase the risk of injuries (both total and severe) during heat waves. Small companies had a proportionately higher share of severe injuries during heat waves and higher costs. Higher cost of severe injury for specific agencies of accident (structure, electricity, environment, small tool, and vehicle).
Spector et al. (57)	Descriptive study of heat-related illness	Washington, US	Maximum and minimum temperature and temperature range, heat index	Agriculture and forestry sector (84 heat-related claims in the study period)	1995–2009	Cost per compensation claim (USD). Time-loss days per claim measured as lost worktime due to work-related injury or illness after a 3-day waiting period (days)	Descriptive analysis of determinants of heat-related compensation claims	Comorbidity and drug use increase risk of heat-related claim. The mean Tmax for outdoor agriculture and forestry heat-related injuries claims was 95°C (99°C for Heat Index). 76% of agriculture and forestry heat-related injuries in males. The mean cost per heat-related claim was 3502 USD and 3071 USD for total and non-compensable (medical-only) claims, respectively. Mean number of time-loss days was 25 (0–96) days. Costs were several times lower than average cost of all claims (not only heat-related ones and in all sectors). Severe heat-related claims (requiring hospitalization or death) mean cost was 24,533 USD.
**Studies evaluating healthcare-related costs**								
Xiang et al. (58)	Time-series study on heat-related workers compensation claim data for injuries	South Australia	Maximum temperature	All work sectors (438 heat-related occupational injuries in the study period)	2000–2014	Work days lost due to injury Medical costs of heat-related injuries (Australian dollars)	The quantitative association between heat and work days lost (count data) assessed using negative binomial regressions to account for over-dispersion	A 1°C increase in Tmax above about 33.8°C was associated with a 41.6% increase in medical costs and a 74.8% increase in days lost due to OHI, respectively. Average expenditure per claim is larger in males, in 25–44 age group, in new workers, in medium size employer, in advanced clerical and service workers, in mining sector, in workers not having had injuries before. There were no significant differences in medical costs and work days lost between heatwave and non-heatwave periods.

PPE, personal protective equipment; BMI, body mass index; USD, US Dollars.

### 3.1. Field studies

Most field studies (20 out of 32) were conducted in low- or middle-income countries (18, 20–22, 24, 27, 29–31, 33, 37, 38, 40–42, 45–47, 49), with only 11 studies from Europe, the USA, and Australia/New Zealand (19, 23, 25, 26, 28, 34, 35, 39, 43, 44, 48) and 1 multicenter study (36). Three studies were qualitative, based on interviews or focus groups (28, 33, 43), while the other studies were quantitative with 27 cross-sectional and 2 longitudinal studies (26, 42) and provided an estimation of the association between heat and labor productivity measured in the field or perceived by workers. The study size overall ranging from 16 (30) to 4,095 workers (21) in different occupational sectors (9 on agriculture, 4 on construction, 1 on mining, and 18 from several sectors) also includes indoor workers (13 studies). Two main approaches were followed by studies in estimating heat impacts on productivity. The first approach provides an estimation of productivity and then evaluates the losses due to heat by comparing productivity data collected at different WBGT levels. In these studies, productivity was estimated in different ways: by worktime (direct, indirect, and non-productive) (22, 30), cognitive and physical performance (e.g., time to complete task/work extra hour absenteeism/taken sick leave) self-reported from questionnaires or interviews (26, 30, 32, 38), clinical examinations (e.g., walking speed) (42), daily output reported or measured by field instruments (e.g., tally counters) (21, 25), and premature worker attrition (21). In the second approach, productivity was not measured by itself, but in terms of productivity losses due to heat in different ways: published physiological models (31), self-reported by workers (29, 49), and prediction models of economic losses due to heat based on number of laborers and the given laborers salary when exceeding WBGT thresholds (44). All studies used individual productivity measures except Amini et al. (18), which evaluated productivity at area level.

Despite the great heterogeneity in the work sectors and study size, nearly all field studies consistently showed a reduction in productivity due to occupational heat exposure. The only exception was one study on office workers not providing evidence of influence of thermal stress on work performance, possibly due to the fact that the thermal stress variable evaluated included both heat and cold temperature; therefore, their single contributions on work performance could not be disentangled (26). The estimated productivity losses ranged between 0.3% and 10% reduction for an increase of 1°C in WBGT (30, 40–42, 47). Considering the whole summer season, the prevalence of workers reporting heat-related productivity loss varied among studies from 11% (46) to 81% (35). Some studies also found an association of heat with an increase in indirect non-productive time at work (30), an increase in idle time at work (30), or in personal household time needed to rest to adapt to heat stress (22). Four studies (36, 44, 48, 49) also provided an estimate of the related economic costs by applying the productivity losses to the gross wages or income of workers with an estimated cost of 6–8 euros per hour worked in Italy (36), 1100 Canadian dollars annually per worker in Ontario (44), 655 USD annually per worker in Australia (48), and 257 euros annually per worker in Malaysia (49). In one study (45), 25% of the workers self-referred a loss in their wages due to fatigue or sickness related to heat. The study by Langkulsen et al. (27) showed a reduction in productivity only in two of the occupational sectors considered (pottery and construction) but not in the others. Only Li et al. (30) and Yi and Chan (47) adjusted for individual worker characteristics such as age and BMI. Given the cross-sectional approach adopted in most studies, the results do not allow causal inference on the association between occupational heat exposure and work productivity.

In some field studies, specific worker subgroups appeared to be more susceptible to the productivity losses due to occupational heat exposure: men (38, 40, 45, 48), females (31), younger, less educated or less experienced workers (37), workers exposed to direct sun (36), workers performing heavy tasks (45, 48), those using personal protective equipment (PPE), such as face masks (23, 29, 35), those affected by comorbidities such as kidney failure or other conditions (21, 22), migrant workers (32, 34), and workers not following safety protocols such as hydrating or taking breaks in cooling places (20, 24, 37).

### 3.2. Studies evaluating healthcare-related costs

In contrast to the field-based studies, the eight studies estimating healthcare-related costs due to occupational heat exposure used data from administrative databases; therefore, they were mostly conducted in western countries such as Europe, Australia, the US, and Canada (50–52, 54, 56–58), with only one study from China (53). Six studies considered all occupational sectors, while three studies only considered specific sectors, such as agriculture and construction. Five studies were descriptive analyses of occupational injuries or diseases identified as heat-related and consequent compensation costs in specific occupational sectors (50–52, 56, 57), while the other three were etiological studies estimating the occupational injuries attributable to heat exposure through time-series or case-crossover analysis and then quantifying the related costs (53, 54, 58).

The national Spanish study from Martinez-Solanas et al. (54) was the only one to estimate heat-related injuries corresponding costs including not only the direct costs attributable to social or private insurance refund to the workers (for long-term losses) or to the healthcare system but also the indirect costs associated with maintaining production, and costs of pain and suffering. The total economic impact of heat-related injuries in the study period was 320 million euros, with the costs associated with pain and suffering higher than other types of costs. The study conducted by Ma et al. (53) in China evaluated the attributable fraction of insurance pay-out related to occupational heat exposure (temperatures above the limit of the Wet Bulb Globe Temperature (WBGT) in accordance with international standards) of 4.1% (95% CI 0.2%–7.7%). In an Australian study, an increase of 1°C in maximum temperature above 33.8°C was associated with an increase of 41.6% in healthcare costs and 74.8% in working days lost due to heat-related injuries (58).

Two descriptive studies conducted in Washington State, US (50, 52), reported an increase in both the median cost per heat-related injury over time (from 537 USD in 1995–2005 to 909 USD in 2006–2017) and the number of working days lost due to injury (from 46 to 93 days per claim on average). The studies also reported an increase in temperatures over time associated with the injuries. In the same study area, Spector et al. (57) estimated a median cost per claim of 654 USD specifically for the agricultural and forestry sectors similar to the previous study conducted in the area (50). These studies suggest a higher median costs related with non-compensable claims for heat-related than for total injuries suggesting a possible under-reporting of work-related accidents in this sector (50, 52, 57). Another South Australian study on construction industry (56) calculated average cost during heat waves higher than in control periods (26,381 vs. 12,747 Australian dollars), with higher costs in the urban area than in the suburbs and for specific agents of injury (i.e., work platform, electricity, and equipment). Finally, a Canadian study in Ontario (51) estimated the rate of injuries related to loss of productive worktime (injury loss time), which is equal to 1.7 cases per million full-time equivalent months in the period 2004–2010.

Studies estimating healthcare-related costs identified some worker subgroups are related to higher costs or worktime losses such as manual workers (51), Black or Latinos workers (52), new workers (56, 58), workers aged 15–24 years (51), men (53), and workers of small- (56) or medium-sized companies (53, 58).

### 3.3. Results from economic studies

Table 2 describes the results of the 49 economic studies at the global (59–70, 72, 74–80) and regional level (73, 81–107). Most studies focused on impacts of current and projected heat on workers' productivity, with the exception of some studies estimating production output reductions due to heat (59, 72, 83) or studies evaluating heat-related impact on both workers and farm production output (66, 70, 100, 101). Climate change scenarios were considered in most studies, with only few studies providing estimated economic impacts for current climate only (66, 73, 77, 78, 91, 97, 98, 100, 101, 105). Studies based on economic models have used different approaches to estimate the economic costs associated with heat-associated reductions in worker productivity. The majority of studies starts from the working time losses estimated based on occupational health and safety standards at different work intensities and sectors. Most included studies estimated productivity as a function of the ISO 7243 standard on the risk associated with thermal stress, by considering exceeding a threshold of the Wet Bulb Globe Temperature indicator (WBGT) at the workplace or on the basis of the standard of indoor thermal comfort, the Predicted Mean Vote Index, and associating climate data with economic data or on the basis of previous studies (e.g., Hothaps models) (65, 67, 69, 91, 108). Worktime losses per day/worker are then rescaled to the entire worker population and expressed in terms of percent productivity loss, converted into monetary terms (e.g., by multiplying for average wages) or as portion of gross domestic product (GDP) considering the share to which each labor sector contributes to GDP. For studies on climate change scenarios, the incremental change relative to baseline is estimated compared to future scenarios usually at the middle (2050) and the end of the century (2100) comparing low and high emissions scenarios. With regards of studies focusing on farm production output, some of them applied structural economic models based on the so-called computable general equilibrium (CGE) model or general equilibrium models that allowed to consider the relationships and influence between economic sectors (70, 73, 74, 87, 97, 105). General equilibrium models are a class of economic models that use actual economic data to estimate how an economy might react to changes in policy, technology, or other external factors (109). Other studies quantified production output using empirical or literature data (66, 72, 100, 101). Some studies included in the economic modeling also an adaptation measure such as air conditioning for indoor work, or shifting work hours or lightening workloads (83, 84, 91, 94, 103, 104).

**Table 2:** Results of economic modeling studies estimating productivity, social, or economic losses related to occupational heat exposure present and future at the regional and global level.

**References**	**Country**	**Heat exposure**	**Work sectors**	**Study period**	**Productivity or cost calculation (unit measure)**	**Heat-related economic loss analysis**	**Results**
**Global studies**							
Burke et al. (59)	Global and regional level (rich and poor countries)	Annual mean temperature	Several occupational types	2050–2100 compared to 1960–2010 (two socioeconomic scenario consistent with RCP8.5)	Productivity of industries not of individuals (change in GDP per capita)	Econometric study. Non-linear analysis between global and country economic production and temperature. Industry productivity is a function of capital and labor and respective productivities that are influenced by temperature. To form a measure of aggregate output, such as gross domestic product (GDP), industry-specific productivity is summed up across all industries and integrate production across all locations in a country and all moments in time within the period of observation.	Overall economic productivity is non-linear in temperature for all countries, with productivity peaking at an annual average temperature of 13.6°C and declining strongly at higher temperatures. Climate change reduces projected global GDP by 23% in 2100 (best estimate, SSP5) relative to a world without climate change. Reductions are similar in rich and poor countries, while are larger in countries becoming warmer.
Chavaillaz et al. (60)	Global and regional level (high- and low-income countries)	Wet-bulb Globe Temperature (WBGT) index of heat stress	vulnerable industries to heat exposure (agriculture, mining and quarrying, manufacturing, and construction workers)	Different emission scenarios (1% CO_2_, RCP4.5, and RCP8.5) compared to the pre-industrial period (1861–1880)	Productivity calculated as working time (resting times)	Estimation of the effect of heat exposure on working time loss per year (due to increasing resting times) based on WBGT safety threshold for different job intensities. From statistics of the International Labor Organization, the change in annual labor productivity (expressed as a relative annual loss of total GDP) for each country and each vulnerable economic sector was calculated based on mean hourly output of the sector, and the GDP of the country. Socioeconomic conditions assumed to be invariant over the study period.	The relationship between productivity loss and CO_2_ emissions is robustly linear at global scale. For each trillion tons of carbon emitted, the annual productivity loss will globally increase by 1.84% (±0.94, 1σ-intervals due to climate and inter-model variability), 2.96% (±1.97) and 3.61% (±1.77) of total GDP in the 1% CO_2_, RCP4.5 and RCP8.5 scenarios, respectively. Some high middle-income countries are subject to the highest impacts; for example, Gabon, India, Thailand, and Malaysia all experience productivity losses from 3 to 5% of total GDP per year for every TtC emitted. Non-CO_2_ gases contribution seemed larger than that of CO_2_ alone.
DARA (61)	Global and country level	Annual mean temperature	Several occupation types (both indoor and outdoor)	2030 scenario SRES A2 vs. 2010 (baseline)	Work capacity as the maximum percentage of an hour that a worker should be engaged working (%) (62)	Analysis of labor productivity losses from WBGT change exceedance of safety standards in acclimatized populations. The changing structure of the workforce over time, in particular, the industrial shift of developing countries away from outdoor agriculture was included. Assuming the different work intensities for each sector, regional labor productivity change estimated based on the weighted average of work activities across each sector. Labor patterns assumed to change over time, consistent with economic growth projected under the A2 emission scenarios.	These results projected a total global GDP loss of US$2.5 trillion (PPP $) per year for 2030 (1% loss of global GDP in 2030, 0.5% loss in 2010). As a percentage of the national GDP, losses varied markedly and were greatest in tropical low- or middle-income countries (e.g., 0.0% in the United Kingdom and Japan, 0.2% in the United States, 0.8% in China, 3.2% in India, 6.0% in Indonesia and Thailand, and 6.4% in Nigeria and Ghana)
Dasgupta et al. (63)	Global and regional level	Mean temperature and wet-bulb globe temperature (WBGT).	Low-exposure working conditions (labor outside in the shade or indoors—e.g., manufacturing) and high-exposure working conditions (outside with no shade—e.g., agriculture and construction)	1.5°C, 2.0°C, and 3.0°C of global warming compared with the historical baseline period (1986–2005)	Change in Effective Labor = (100% + Change in Labor Supply) ^*^ Change in Labor Productivity, where labor supply is measured by hours worked and labor productivity derived from published temperature-productivity functions	The effect of climate change on labor productivity was assessed using five different exposure-response functions established in the literature. Warming levels were assessed over the observational period until the reference period 1986–2005 (0.6°C) and modeled warming for the individual climate models relative to the reference period.	Current climate conditions already negatively affect labor effectiveness, particularly in tropical countries. Future climate change will reduce global total labor in the low-exposure sectors by 18% and 24.8% in high-exposure sectors under 3°C global warming scenario. Higher impacts for labor outdoors in full sunlight. Europe is expected to be the least affected region, while the highest impact will be in Sub-Saharan Africa.
De Lima et al. (64)	Global and regional level	WBGT	Agriculture	1.5°C, 2.0°C, and 3.0°C of global warming compared with the 1986–2005 baseline	Individual labor capacity estimated (1) by WGBT safety standard (NIOSH) for agricultural workers (400 W) and an associated function for labor capacity; and (2) by Dunne algorithm (65)	Labor capacity change estimated for baseline and climate change scenarios accounting for impacts in crop yields change. Global economic model to predict impacts on yield and labor changes.	In sub-Saharan Africa and Southeast Asia heat stress with 3°C global warming could reduce labor capacity in agriculture by 30%−50%, increasing food prices and requiring much higher levels of employment in the farm sector.
Dunne et al. (65)	Global level	Wet-bulb Globe Temperature (WBGT)	Outdoor workers	Reanalysis 1971–1980 and 2001–2010, projected 2091–2100 and 2191–2200 under high emissions (RCP 8.5) and mitigation (RCP 4.5) scenarios	Population-weighted individual labor capacity (%) during annual minimum and maximum heat stress months estimated from WBGT applied to US national and international standards for safe work intensities (90% means 10% losses in labor capacity)	Analysis focused on the loss of labor productivity as a function of WBGT levels during the hottest months in reference period and under scenarios.	Reductions in work capacity during the hottest months already occur at the global level (10% reduction). By 2050 under both scenarios, work capacity loss is 2-fold higher than in the historical period (20% reduction). By 2100, the reductions in the hottest month may reach 37% based on RCP8.5 and 20% based on RCP4.5. By 2200, very significant further changes in work capacity are projected for the hottest month based on RCP8.5 (61% reduction), and 12% of population is exposed to work capacity losses. To offset these reductions a substantial increase in unskilled farm workers will be required.
Heal and Park (66) Gray literature	US and other countries at global level	annual mean temperature	all work sectors	1950–2005 no future climate change scenarios	Output shocks related to heat as % per capita GDP per 1°C (Per capita output is increasing in effective labor supply)	Analysis based on statistical model of work output (or GDP) and thermal stress (controlling for institutions, capital stock, and education). Linear regression between GDP is produced using a combination of capital and effective labor input. Effective labor input is defined as a composite of labor hours, labor effort, and labor performance as a function of the ambient temperature. We allow for the possibility that temperature may affect GDP with a time lag, by allowing for 1, 5, and 10 lags. For the US, household data on air conditioning and heating expenditures.	Very hot countries such as Thailand, India, and Nigeria suffer negative output shocks on the order of 3–4% per capita GDP per 1°C. Very cold countries such as the UK, Canada, Norway, and Sweden have significantly higher output in warmer years (and lower output in colder years). In the US, a household with an average age of 20 spends roughly 15% (28 USD) more per year on air conditioning and 12% (54 USD) less on heating than an otherwise equivalent household with an average age of 60 and expenditure on both air conditioning and heating are higher for households with someone at home who is working than for those with someone at home but not working.
Kjellstrom et al. (67)	Global and regional level (21 geographical regions)	Wet-bulb Globe Temperature (WBGT)	All work sectors (service, industry, and agriculture) both indoor and outdoor	2020, 2050, and 2080 compared to 1961–1990 under climate scenarios SRES A2 (worst) and B2 (best)	Change in labor productivity expressed as percent work days lost and incremental change relative to baseline.	Projections of future labor productivity losses (in terms of lost labor days) under climate scenarios compared to baseline climate applying dose–response function between WBGT and work capacity estimated in Kjellstrom et al. (67). Assuming the different work intensities for each sector, regional labor productivity change estimated based on the weighted average of work activities across each sector. Labor patterns assumed to change over time, consistent with economic growth projected under the A2 emission scenarios.	By the 2080s, the greatest absolute losses of population-based labor work capacity (in the range 11% to 27%) are seen under the A2 scenario in Southeast Asia, Andean and Central America, and the Caribbean. Under B2 scenario smaller impacts in all regions (the greatest loss being 16% in Central America), and labor productivity gains in some regions (up to 6%).
Kjellstrom et al. (68)	21 global regions (high and low- and middle-income countries)	Wet-bulb Globe Temperature (WBGT) index of heat stress (calculated using Hothaps functions)	All work sectors (outdoor and indoor)	2030 and 2050 vs. 1960–1989	Reduction of hourly active worktime expressed as loss of work capacity due to heat. Cost of labor productivity loss due to excessive heat, % of GDP	Lost work capacity calculated using exposure–response relationships from literature. The national loss estimates used the proportion of the work force in jobs with different physical demands and different heat exposure levels, based on a World Bank model. The losses, as percent of daylight work hours, were multiplied with the estimated GDP for 2011 and 2030. Workforce changes are taken into account.	For Southeast Asia work capacity losses increase from 17% to 29% (of daylight work hours) from 1975 to 2050 for outdoor workers doing heavy labor. The losses for indoor workers doing heavy labor increase from 3% to 8%, and for outdoor workers doing moderate labor the estimates go from 7% to 15%. Low- and middle-income countries have losses 6% of annual GDP, higher compared to high income countries. The estimated annual losses, expressed as $US PPP, are already in 2010 up to 55 billion (India) and in 2030 up to 450 billion (India and China).
Kjellstrom (16) (gray data embedded in the review)	global and country level	Wet-bulb Globe Temperature (WBGT) index of heat stress	all work sectors (service, industry, and agriculture) both indoor and outdoor for a mixed workforce (average metabolic rate = 300W; in shade or indoor non-cooled work)	2085 (2070–2099) under global warming scenarios 4°C (RCP8.5) (worst) and 1.5°C (RCP 2.6) (best) compared to 1995 (1980–2009)	Person-hours lost due to heat in each region (i.e., the work capacity loss multiplied by the working population in each grid cell and then summed up for all grid cells in a region). Lost work hours are expressed as the annual percent of daylight hours lost due to heat at 300W.	Projections of future labor productivity losses (in terms of lost labor days) compared to baseline climate, applying dose–response function between WBGT and work capacity estimated in literature for moderate labor	Productivity is already lost up to 10% of annual daylight hour in some regions. There is a 10-times or more increase of work hours lost from 2015 to 2085 for several countries under RCP8.5 scenario. The substantial reduction in work capacity (and related labor productivity) between 1995 and 2085. The areas with the greatest risk in 2085 remain the same (Amazon region, West Africa, Arab Gulf area, Pakistan, North India, Indonesia, and parts of China), but substantial reductions in work capacity are apparent in the southeast United States, parts of Europe (South), Africa, and the rest of India and China. By the end of the century impact will increase in the hottest areas even if temperatures are held at 1.5°C (RCP2.6), but the increase is much higher for the business-as-usual scenario of 4°C (RCP8.5), reaching more than 30–40%.
Kjellstrom et al. (69)	Seven countries (USA, China, India, Cambodia, Philippines, Ethiopia, Costa Rica)	Wet-bulb Globe Temperature (WBGT) index of heat stress	all work sectors (service, industry, and agriculture) assuming agriculture is the hardest work (400 W) and mostly occurs outdoors in the sun, industry at 300 W is in the shade or indoors and servicing the easiest at 200 W and in air conditioned spaces	2011–2040, 2041–2070, and 2071–2099 vs. 1981–2010 under climate change scenarios of 1.5°C (RCP2.6) and 2.7°C warming (RCP6.0)	Percent of potential work hours lost calculated from daylight person hours lost for each grid cell in a geographical area and total potential work hours in the population.	Productivity loss due to heat estimated based on dose–response function between WBGT and work capacity in each grid cell and each region. Projections of future labor productivity losses estimated for workers in moderate-intensity jobs (MR = 300W).	Under the more extreme climate change trend (RCP6.0; increase of 2.7 °C), as much as 12–16% of annual work hours will be lost in some areas. The impact is naturally mainly occurring in the southern hotter areas. Countries with large cool climate areas (such as USA) have limited work hour losses due to heat now (0.17%), but it may increase beyond 1.3% at the end of the century based on the current global climate policy pathway (RCP6.0). The most affected countries, such as Cambodia, may have losses exceeding 10%.
Kjellstorm et al. (62) gray (ILO report)	global and regional level	Wet-bulb Globe Temperature (WBGT) index of heat stress	all work sectors (agriculture, construction, industry, services)	2030 compared to 1995 (1981–2010) under RCP6.0 (worst) vs. RCP2.6 (best)	Estimated annual labor productivity losses expressed as total working hours, or $US PPP (or % of GDP) or equivalent full-time jobs due to excessive heat	Productivity loss due to heat estimated based on dose–response function between WBGT and work capacity in each grid cell and each region for moderate and heavy labor. Projections of future labor productivity losses estimated for workers in moderate-intensity jobs (MR = 300W).	By 2030 the share of total working hours lost will rise to 2.2%, a productivity loss equivalent to 80 million full-time jobs at global level. The loss in monetary terms is then expected to total US$2, 400 billion (PPP). Lower-middle- and low-income countries would be the worst affected, losing 4% and 1.5% of their GDP in 2030, respectively. Losses are close to zero in Europe. Agricultural and construction workers will be the worst affected. The agricultural sector alone accounts for 83% and 60% of global working hours lost to heat stress in 1995 and 2030, respectively. Construction is expected to account for 19% of the total loss in 2030, up from 6% in 1995.
Knittel et al. (70)	All world regions (European and not European)	Wet Bulb Globe Temperature	heavy outdoor work (agriculture, construction) and medium intensity indoor work (manufacturing industry)	Shared Socioeconomic Pathways (SSP1, SSP2, and SSP3) and two Representative Concentration Pathways (RCP4.5 and RCP8.5) in 2036–2065 vs. 1981–2010	Productivity loss as relative change in work ability (%). Productivity costs as output losses due to excessive heat, % of GDP	Impact of heat on production estimated based on heat-production function by literature (71) based on labor input, change in work ability based on dose–response function between WBGT and work capacity from literature. The study evaluated these impacts on Germany economy using a global CGE models.	Impacts on productivity higher for outdoor than indoor work. By 2050, within Europe, reductions are most pronounced for Italy and other Mediterranean countries (Cyprus, Greece, Malta, Portugal, Spain), while other countries are only marginally affected. Other world regions are severely impacted such as Southeast Asian countries, India, and oil exporting countries. In the Amazon region, heavy outdoor work (400W) is projected to decline by more than 50% under RCP8.5.
Kuhla et al. (72)	global and regional level	daily mean temperature	agriculture, fishing, mining and quarrying, hotels and restaurants, wholesale trade, and others	2020–2039 vs. 2000–2019 (RCP2.6 and RCP6.0 scenarios)	Direct output production losses by region (billion USD). Also indirect production and total losses (in terms of value of goods and services) are calculated.	Absolute and relative heat stress-induced direct output losses based on risk function between temperature and productivity from literature (perturbed productivity) (73). Absolute output losses are then determined by multiplying the perturbed productivity with the baseline production of that region	Globally, between 2000 and 2039 direct output losses increase by 47% if no further adaptation measures are taken. Regional increase in direct losses in the billions USD (e.g., in India, Saudi Arabia, or Mexico) or nearly double the direct output losses (e.g., in Northern America or Europe) within the next decades.
Matsumoto et al. (74)	global and regional level	Wet-bulb Globe Temperature (WBGT)	agriculture, manufacturing, and service	2100 business-as-usual scenario BaU, and two emission-reduction scenarios (“S45” and “S2”) vs. 2007 (baseline)	labor productivity reductions (%) and associated GDP losses (%)	Coupled socioeconomic (CGE) and climate models. Changes in labor productivity affect the labor input necessary to produce goods/services in the production functions. Climate change impact on labor productivity based on dose–response function between WBGT and work capacity estimated in literature.	The impacts were the largest for the agricultural (36.8–100% labor productivity reduction by 2100), and the lowest for the service sectors (83.0–100% productivity reduction by 2100). Labor productivity reached its minimum levels during the warmest and wettest parts of the year in already hot and humid regions (similar trends were observed for both of the mitigation scenarios as well). Such declines in labor productivity reduced production and, consequently, affected the macroeconomy. The global-level negative impact on GDP grew with temperature increases, which was about 2% per 1°C
Orlov et al. (75)	Global and regional level	Wet-bulb Globe Temperature (WBGT)	all work sectors agriculture and construction (high-intensity jobs, 400 W), manufacturing and services (moderate-intensity, 300 W and low-intensity work, 200 W)	2020, 2030, 2040, 2050, 2060, 2070, 2080, and 2090 compared to 1981–2005 under RCP8.5 (worst) and RCP2.6 (best) scenarios (and SSP1, SSP4, and SSP5 scenarios for CGE model)	Percentage reductions in global GDP from labor productivity loss, estimated by decreased work efficiency	Interdisciplinary approach that combines climate projections, epidemiological findings, and economic analyses. Work capacity loss (a physiological variable) estimated using the dose–response function between WBGT and work capacity estimated in literature (Hothaps and ISO). The spatiotemporal data of relative worker productivity losses are matched with the gridded data on the population count to obtain population-weighted impacts on worker productivity at a regional level. and the associated economic costs are assessed by using a dynamic multi-region, multi-sector computable general equilibrium model. Autonomous mechanization of outdoor work in agriculture and construction and presence of air conditioning for indoor work is implemented in the model.	Heat stress leads to substantial reductions in worker productivity. For RCP8.5, using the Hothaps function with constant work intensity results in an average reduction of 0.7% (1.8%) in global GDP by 2050 (2100) relative to the reference period. Impacts are higher for high-intensity work in low-latitude countries of Africa, South America, and Asia. Given the assumption of absence of air conditioning and constant work intensity, reductions in worker productivity in some regions under RCP8.5 could even exceed 40% by 2100 compared to the reference. Approximately 42% of the global mitigation cost could be offset by avoiding the adverse impacts of heat on worker productivity. Agriculture and construction are the most adversely affected by heat because these sectors require many work-intensive activities in the outdoor environment. While many low-latitude regions experience considerable reductions in worker productivity, less vulnerable regions such as Oceania, North America, Former Soviet Union, and Europe, receive a comparative advantage in production of agricultural goods, which explains those moderate increases in their production. Due to the penetration of air conditioning and a lower work intensity, manufacturing is less adversely affected by heat compared to agriculture and construction. The service sector exhibits a low risk of exposure to heat.
Parsons et al. (76)	global and country level	WBGT	outdoor heavy labor work sectors (agriculture, forestry, fishing, and construction)	2001–2020 and future scenarios (1°C, 2°C, 3°C, and 4°C climate warming)	Heavy labor lost (hours) Productivity loss (Billions PPP USD)	Models combining climate projections and epidemiological findings on heat-productivity risk functions. The spatiotemporal data of work capacity loss based on WGBT using the dose–response function between WBGT and work capacity estimated in literature (at 400W intensity) in the 12-h work day and combining with the working population in each country to estimate the heavy labor work hours lost. Economic losses related to lost earnings were also calculated by estimated of hourly earnings and converting hours lost to job loss equivalent and expressed as reduction in GDP.	Strong relationship (non-linear) between global annual mean temperature and annual sums of hours lost both in the reference period and in future global warming scenarios. In the current climate, 25–30 h lost/person/year could be recovered if workers in many low-latitude regions could move heavy labor to a cooler hour from the hottest hour of the day. Current global estimates of productivity losses are 670 billions PPP USD in the 12-h work day every year. Under +2°C warmer world, productivity losses reach 1.6 trillion PPP USD.
Parsons et al. (77)	global and country level	WBGT	outdoor workers in heavy labor sectors (agriculture, forestry and fisheries; construction)	2001–2020 compared to 1981–2000 (baseline) (no climate change scenarios)	Heavy labor lost (hours) Productivity loss (Billions PPP USD)	The spatiotemporal data of work capacity loss based on WGBT using the dose–response function between WBGT and work capacity estimated in literature (at 400W intensity) in the 12-h work day and combining with the working population in each country to estimate the heavy labor work hours lost. Economic losses estimated by multiplying the full-time equivalent (FTE) work hour loss by the average value added per worker in each sector. To calculate FTE work hour losses, we divide the hours lost per year by the total possible work hours in a year by sector and region, expressed as GDP per capita (PPP) in USD.	Over the study period, global-mean, near surface air temperatures have increased by ca 0.4°C resulting in increases in per capita labor losses of up to 150 h person−1 yr−1 (12.5 days person−1 yr−1) in some low-latitude regions. Global labor losses higher estimates are 2.1 trillion PPP USD. China and India again experiencing the largest losses, and Indonesia and the United States showing over 90 billion PPP USD losses per year. India experiences annual productivity losses equivalent to almost 7% of its 2017 GDP.
Romanello et al. (78) (the Lancet Countdown 2022)	global and regional level (low, medium, high, and very high human development index)	Wet Bulb Globe Temperature	agricultural, construction, manufacturing and service sectors	1990–2021 (annual estimates)	Potential hours of labor lost due to exposure to heat by labor sector (in millions)	Hours of work lost calculated by linking Wet Bulb Globe Temperature with the amount of energy typically expended by workers by sector and combining with the proportion of people working (over 15 years old) in each country.	470 billion h of potential work were lost due to extreme heat exposure in 2021, —an increase of 37% from the annual average in 1990–99, an average of 139 h lost per person, with 87% of all losses in countries with a low Human Development Index occurring in the agricultural sector. Two-thirds of all labor hours lost globally in 2021 were in the agricultural sector. Conservative estimates since shade work is considered.
Roson and Sartori, (79)	global and regional level	Wet-bulb Globe Temperature (WBGT)	Agriculture (high intensity, 500W), manufacturing (medium intensity, 300 W), service (low intensity, 200 W)	Global warming scenarios of 1°C, 2°C, 3°C, 4°C, and 5°C increases in average temperature (study period not specified)	Relative percentage change in (annual) productivity with respect to the baseline, for all countries and sectors	The spatiotemporal data of work capacity loss estimated based on WBGT using the dose–response function between WBGT and work capacity found in the literature projection of loss in labor productivity from relationships between average temperature and labor productivity under scenarios of 1, 2, 3, 4 and 5 °C increases in average temperature (study period not specified).	The estimated percentage variation of labor productivity for 140 regions and for a +1°C increase in temperature is−0.27%. The mean productivity losses range from−2.52% to−17.48%. Agriculture is the sector most significantly affected by higher heat stress. Some effects are felt by about half of the countries already at +1°C.
Takakura et al. (80)	Global and regional level	Wet-bulb Globe Temperature (WBGT)	all work sectors (outdoor and indoor) industry and construction (high intensity, 400W), manufacturing (moderate intensity, 300W), and service (low intensity, 200W)	2100 under four representative concentration pathways (RCP2.6, RCP4.5, RCP6.0, and RCP8.5) and three socioeconomic scenarios (SSP1, SSP2, SSP3) compared to baseline (2005)	Work–rest ratio changes (worktime reductions) Productivity losses (worktime lost) and direct costs of worktime loss both expresses as GDP percentage reduction under climate change scenarios compared to the reference period	Work capacity (work hours loss) estimated based on WBGT using the dose–response function between WBGT and work capacity found in the literature and on safety recommendation of work/rest ratio. Daily total worktime was calculated by the hourly work capacity and summed the hourly work capacity from 9:00 AM to 5:00 PM. In order to express the labor productivity loss due to reduced worktime in economic costs, the labor input was multiplied by the ratio of the worktime reduction, and their product was used as the effective labor input to the production function. The direct cost is calculated as the additional wages required to compensate the worktime loss associated with the additional labor requirements. Presence of air conditioning for indoor work is implemented in the model.	At the end of the 21st century, the aggregated worktime ratios decrease under both low and high emission scenarios. Under RCP8.5, the aggregated worktime ratios decrease to 0.23 in Southeast Asia, 0.36 in India, and 0.42 in Sub-Saharan Africa. Indoor work is also adversely affected under RCP8.5. For example, in India, the worktime ratio was 0.62 for indoor/moderate work and 0.76 for indoor/light work if air-conditioning devices are not available. Under the highest emission scenario, GDP losses in 2100 will range from 2.6% to 4.0% compared to the current climate conditions. Under RCP8.5, the GDP loss rates (median values) are 14.3%−17.3% in India and 4.6%−6.9% in Southeast Asia. In terms of direct costs, the construction sector is affected primarily by worktime loss in terms of direct costs. Based on the relationship between temperature increase and GDP loss, if the 1.5°C goal were achieved, the GDP loss rate would be reduced by approximately 0.3%, as compared to that of the 2.0°C goal.
**Regional studies**							
Altinsoy and Yildirim (81)	western Turkey	WBGT	agriculture and construction at different work intensities (light, medium, heavy, very heavy)	1971–2000 (baseline) 2011–2040, 2041–2070, 2071–2100 (scenario SRES A1B - one of the highest emissions scenario) Only Spring, Summer, Autumn seasons.	labor productivity losses in terms of percentage of potential work days in season	Spatiotemporal WGBT values used to calculate labor productivity losses as work hour loss estimated from recommended rest/work ratio (25%−50%−75%−100% corresponding to 15–30–45–60 min rest for 1 work hour) at different WBGT values and work intensities. Expected decline in labor productivity is multiplied with the agriculture contribution to the economy to yield the total decline in labor productivity in agriculture.	The most important productivity decreases are expected in the summer. The main impact on work productivity becomes evident after 2040. In Turkey decrease in labor productivity losses in agriculture vary from 1% (baseline), to 2% in 2011–2040, 5% in 2041–2070, and 8% in 2071–2100. In some areas, the largest decrease reaches 52%.
Amnuaylojaroen et al. (82)	5 megacities in Thailand	Steadman Heat Index	not specified	1990–1999 (baseline), 2020, and 2029 RCP 8.5 (very high emissions)	Percent decrease in labor productivity (%)	Labor productivity losses (work hours) calculated from heat index with a formula based on experimental data	The maximum decrement in work performance there was in December between 4% and >10%, with Southern areas facing most decrement than the rest of the country.
Behrer and Park (83) Gray literature	US	Daily maximum temperature	Non agricoltural sectors	1986–2011 and climate change scenarios in 2040–2050 (under RCP 4.5)	Annual payroll per capita (close proxies to changes in total and marginal labor product)	Analysis of heat stress impact on production outputs (payroll per capita) based on inputs labor productivity, effective labor supply, and temperature stress (number of hot days per year with maximum temperatures above 95°F). Panel regression of payroll and maximum temperature by county and year. Presence of air conditioning for indoor work is implemented in the model.	An average US county experiences a−0.04% reduction in payroll per capita during a year with one additional day with maximum temperatures above 95°F (35°C). The impacts are roughly 9 times as large in exposed sectors (construction, transportation, utilities, manufacturing, and mining). For instance, lost payroll under a no adaptation scenario is at least 50% higher in 2040–2050 compared a scenario in which local economies adapt to their new (hotter) climates (corresponding to 18 billion USD losses).
Costa et al. (84) Gray literature	3 EU cities Antwerp (Belgium), Bilbao (Spain), and London (United Kingdom)	Wet-bulb Globe Temperature (WBGT)	all work sectors (indoor and outdoor) at different level of intensity	near future (2026–2045) and the far future (2081–2100) scenarios compared to a reference period (1986–2005) (under RCP8.5)	Annual labor productivity loss, estimated by lost hourly worktime, and expressed as % of Gross Value Added (GVA) at the sector level	Production was measured by Gross Value Added (GVA) at the sector level. Spatiotemporal WGBT values used to calculate productivity loss as hourly productivity loss across all working hours and working regimes estimated from recommended rest/work ratio (25%−50%−75%−100% corresponding to 15–30–45–60 min rest for 1 work hour) at different WBGT values (ISO and NIOSH standards) and work intensities. Analysis of sectoral production as a function of WBGT, sector-specific capital and labor. Economic costs estimated by an explicit production function from input capitals and labor for each sector that is aggregated into city-specific Gross Value Added (GVA) at city level. Adaptation measures (shifting work hours, increase in insulation and air conditioning for indoor work) were implemented in the model.	Productivity (annual GVA) loss of 0.4% in London, 2.1% in Antwerp, and 9.5% in Bilbao projected in 2081–2100. These correspond to total losses of around 1, 900 million Euros for London, 669 million Euros in Antwerp, and 2, 500 million Euros in Bilbao, in 2005 prices. Losses will tend to increase with time, in particular in warm years, although not always linearly. GVA was observed to monotonically decrease with increasing WBGT. Losses vary greatly across sectors and by city. The construction sector accounts for only 4% and 6% of losses in Antwerp and Bilbao, respectively, while it accounts for 18% in London. Air conditioning is the most effective in reducing labor productivity losses from heat stress.
deBoer et al. (85) Gray literature	Phoenix area (US)	number of days with maximum temperature over 110°F	all work sectors (both high- and low-risk sectors)	2020–2039 and 2040–2059 (RCP 4.5 low emission and RCP 8.5 high emission) vs. 1986–2005 baseline	Labor productivity losses in million USD and as % of county GRP (Gross Regional Product)	Regression models from literature estimating the relationship between temperature and allocation of time to labor as well as leisure activities (for high-risk labor, time allocated to labor drops by 59 minutes on days with daily maximum temperatures over 100°F). The relative productivity loss was calculated on projected days above the temperature threshold (100°F) relative to the counterfactual in which the number of days above the temperature threshold is equal to the baseline. This number was subtracted from the projected number of days above the temperature threshold (100°F) from projected climate data for a future year. Impacts were estimated both with constant employment and GRP.	Labor productivity losses are 927–1313 and 1512–2138 million USD for RCP4.5 and RCP8.5, respectively, in 2020–2059, corresponding to 0.3% and 0.6% labor productivity losses.
Deloitte (86) Gray literature	Australia	annual mean temperature	all work sectors	global average warming of above 3°C by 2070 under RCP 8.5 compared to 2020 (baseline)	Economic losses due to job losses caused by climate change, as % of GDP or USD	The climate change models for different emission scenarios are the basis for translating a given temperature increase into economic damage by sector, region, and over time. Damage functions include capital damages, sea level rise damages to and stock, heat stress damages on labor productivity, human health damages to labor productivity, agricultural damages from changes in crop yields, tourism damages to net inflow of foreign currency and damages to energy demand. From physical climate damages to the factors of production, then economic impacts are estimated.	The economic losses to Australia from unmitigated climate change are 3.4 trillion USD (2020) or 6% of GDP by 2070. On average over the 30 years to 2050, that is a loss of 135, 000 jobs per year and 1.8% of GDP. The worst impacted industries are service sectors (both government and business), trade and tourism, manufacturing, and mining. Agriculture damages evaluated based on variations in crop yields.
Hsiang (73)	Carribean and Central America	annual mean temperature	different work sectors	1970–2006 by season	change in production due to temperature increases (% change for 1°C increase)	Empirical models of the relationship between the production of value in individual industries and interannual variations in climate. The production of goods and services is measured by per capita value added. Regression models of production with temperature, rainfall, and cyclones evaluated in non-linear models.	Heat impact on total production of−2.5% per 1°C increase. Wholesale, retail, restaurants and hotels (-6.1% per 1°C increase), and other services (-2.2% per 1°C increase) exhibit significant production losses. Output losses occurring in non-agricultural production (−2.4% per 1°C increase) substantially exceed losses occurring in agricultural production (−0.1% per 1°C increase).
Hübler et al. (87)	Germany	perceived temperature (Laschewski, 2002)	all work sectors	2071–2100 (A1B SRES high emission scenario and B1 low emissions) compared to 1971–2000 (baseline)	Average GDP loss per year	Macroeconomic model of the impact of heat on labor output. Predictions of GDP losses for future temperature scenarios are estimated as function of heat-related GDP loss in baseline year (2004), predicted days with temperature exceeding safety threshold, mean relative productivity reduction when temperature threshold are exceeded, GDP in baseline year, wage share in baseline year.	Considering the worst scenario (A1B), future (2071–2100) losses are 2.5 billion Euros (0.12% of GDP) or 10.4 billion Euros (0.48% of GDP) with labor productivity losses of 3% and 12% for strong (32–38°C) and extreme heat (equal or above 38°C), respectively. Actual losses are 540 million Euros and 2.4 billion Euros with labor productivity loss of 3% and 12% for strong and extreme heat, respectively. Using IPCC scenario B1 (low emissions) and a 12% heat impact on labor productivity yields an additional loss of ca. 4.2 billion Euros, which is significantly lower than in the A1B scenario (almost 8 billion Euros, representing the expected emissions development).
Kershaw (88)	UK	predicted mean vote (PMV)	indoor work sectors	2030s, 2050s, and 2080s under SRES A1F1 scenario vs. 1970s	Relative productivity losses (%) Cost of lost productivity per square meter as a result of thermal discomfort	The loss of productivity due to thermal stress for each hour of occupancy is derived from physiological model of productivity and PMV. The cost of lost productivity per square meter as a result of thermal discomfort over the year is estimated based on the productivity per worker within a given sector. This is calculated by dividing the Gross Value Added (GVA) for that sector by the number of people employed in that sector measured as full-time equivalent (FTE). The change in relative productivity as a function of user thermal comfort is then applied to the economic output of a worker. A typical office building is used.	As the climate warms then the cost of lost productivity increases from 134 pounds per square meter in 1970s (3.2% lost productivity) to 148, 164, and 181 pounds (and 3.5%, 3.9%, and 4.3% lost productivity) per square meter in 2030, 2050, and 2080, respectively.
Kjellstrom et al. (89)	Southeast Asia	Wet-bulb Globe Temperature (WBGT)	all work sectors both non cooled indoor (or shade) and outdoor (or sun), for heavy (400W) and moderate (300W) work in the shade and in the sun	1975 (1961–1990) and 2035–2065. No climate change scenarios.	Work loss in percent of available afternoon working hours in March	Projections of future labor productivity losses (in terms of lost labor days) (% loss at specific WBGT level) from dose–response function between WBGT and work capacity for moderate and heavy work estimated in literature and based on safety standard (ISO) function (work hours lost due to rest and slower work due to heat)	In 1975 in the hottest locations 30–40% of afternoon worktime is lost in the shade and 60–70% lost in the sun. In 2050 in hottest areas, afternoon worktime is lost due to heat up to 80% for heavy work and up to 50% for moderate work.
Kopp et al. (90) Gray literature	US	daily maximum temperature	all work sectors for high-risk (agriculture, construction, utilities, and manufacturing) and low-risk labor sectors	2020–2039, 2040–2059, 2080–2099 climate change scenarios (RCP 2.6, 4.5, and 8.5) compared to 2012 baseline	Relative (%) and absolute (full-time equivalent workers at current employment levels) change in labor productivity	Changes in productivity estimated using the dose–response functions between temperature and working time obtained by literature (91). Number of minutes individuals work from survey data. The dose–response functions accounted for cross-county patterns in labor markets, as well as trends over time and over seasons. Dose–response functions were used to predict future changes in labor productivity under different climate scenarios relative to a future with no climate change.	In RCP 8.5, high-risk labor likely declines by 0.2% to 0.9% by 2040–2059 and by 0.8% to 2.4% by 2080–2099. For low-risk labor supply, losses are more modest, with 2080–2099 losses in RCP 8.5 of 0.1% to 0.5%, with a 1-in-20 chance that labor supply falls more than 0.8% or less than 0.01%. Projected changes are smaller in magnitude for RCP 4.5 and RCP 2.6.
Kovats et al. (92) Gray literature	Europe	Wet-bulb Globe Temperature (WBGT)	all work sectors (agriculture, industry, and service) at different work intensities (400W for agricultural labor, 300W for industrial labor, and 200W for service industry)	2020, 2050, 2080 under SRES A1B (medium–high emission) and E1 (low emission) climate change scenarios compared to 1961–1990 (baseline)	Labor productivity losses (in terms of lost labor days) Economic costs (Million Euro/year) related to productivity losses	Changes in productivity estimated using the dose–response functions between temperature and working time obtained by literature (67). Loss of labor productivity, derived from the GDP per labor force member using EU27 average productivity cost value of 287 euros per day. Projections of productivity loss estimated by combining a global temperature rise of 1.5°C by the end of the twenty-first century with labor force trends compared to baseline climate. The model is based on a scenario in which current labor distributions are maintained over time and a scenario in which there is a shift among sectors in Europe.	Under the current climate, the only impacts are in Southern Europe, where losses were estimated to be 0.14% days lost. Higher impacts are projected for Mediterranean countries with climate change. Under A1B scenario, for Southern Europe a 0.4–0.9% loss in productive days by the 2080s. Total productivity losses (whole European area) are estimated at 120–320 million euros in the 2050s, rising to 300–740 million euros in the 2080s under A1B scenario. Impacts are significantly lower under the E1 mitigation scenario. According to the scenario of productivity distributions change, future impacts in Europe are lower.
Lee et al. (93)	South Korea	Wet-bulb Globe Temperature (WBGT)	outdoor laborers in construction, agriculture, forestry, and fisheries (moderate, 234–407 W and heavy intensity, 407–581 W)	2011–2040, 2041–2070, 2071–2100 under RCP 8.5 (high emission) and 4.5 (moderate emission) compared to 1981–2005 (baseline) summer season (June to September)	Labor productivity (days with reduced labor productivity as percentage of total working days) Relative productivity loss in future scenarios compared to baseline.	Projections of future labor productivity losses (in terms of lost labor days) compared to baseline climate, applying dose–response function between WBGT and work capacity (work–rest ratio) estimated in literature for moderate and heavy labor. Population-weighted future labor productivity estimated from the number of days with reduced labor productivity multiplied for reduction ratio expressed as percentage change of the total number of working days. The relative productivity loss was calculated as difference between future and current labor productivity by period.	Future productivity losses for moderate work of 4.8% and 15.8% by 2071–2100 under RCPs 4.5 and 8.5, respectively, compared to the baseline. Productivity losses for heavy work are 12% (RCP4.5) and 26.1% (RCP8.5) compared to the baseline. Areas with larger productivity losses are those with higher proportion of outdoor workers.
Licker et al. (94)	US	maximum heat index	outdoor workers (included agriculture, construction, and transportation) for moderate and light workload	2036–2065 and 2070–2099 (RCP4.5, +2°C and RCP 8.5, +4°C) vs. 1971–2000 (baseline)	Worktime at risk of being lost Annual earnings (Billions USD) at risk (%)	Calculation of the number of hours would be unsafe to work (in terms of lost labor days) based on dose–response function between heat index and work capacity (work–rest ratio) estimated by NIOSH for light and moderate workloads. These findings were coupled with the future annual average number of days projected to exceed heat index thresholds by occupational category and scenario and multiplied by the number of people in each occupational category (e.g., protective service) and refer to this exposure metric as “person-days” per year. Economic losses in terms of earnings at risk (assuming that workers are not paid for the hours during which it is too hot to work) calculated based on unsafe workdays, annual median earnings, and total workdays per year. Two potential adaptation options—using an adjusted work schedule that shifts work hours to cooler times of day and lightening workloads—were also assessed.	Assuming normal work schedules and moderate workloads, nationwide nearly 3 million outdoor workers already experience 7 or more unsafe workdays per year—primarily across Southwest, Southern Great Plains, Midwest, and Southeast. This number will grow by late century to 17.1 million workers (RCP4.5) and 27.7 million workers (RCP8.5). In terms of earning loss 4.7% of earnings (or a total of $49.2 billion) would be at risk under RCP4.5 and 10.2% (or a total of $107.5 billion) under RCP8.5 by the end of the century. Both adaptation scenarios are able to reduce the number of workers at risk, especially the second measure reducing workloads to light levels. By late century, universal implementation of both adaptation measures combined with emissions reductions consistent with the RCP4.5 pathway would reduce the number of workers experiencing 7 or more unsafe workdays per year to virtually none compared with 27.7 million workers who would experience such losses with the higher emissions RCP8.5 scenario and no adaptation measures implemented.
Liu (95)	China	Wet-bulb Globe Temperature (WBGT)	outdoor workers and work intensity for light, moderate, and heavy	near future (2021–2050) and the end of the century (2071–2099) under RCP scenarios 8.5 (high emission) and 2.6 (low emission) compared to baseline (1981–2010) in July and August	Changes in labor capacity (%) Relative productivity loss in future scenarios compared to baseline.	The labor capacity is estimated in terms of lost labor days based on dose–response function between WGBT and work capacity (work–rest ratio) estimated in the literature. Projections of future labor productivity losses compared to baseline climate.	Under RCP8.5, the labor capacity of China would decrease by 5.5–5.6% and 16–17% during the 2021–2050 and 2071–2099 periods, respectively. Large decreases (more than 40%) in labor capacity of heavy work due to increased WBGT were found for many areas of China in future, particularly in northern China especially at the end of the century under RCP8.5 compared to baseline. In South and East China, labor capacity of light work would also experience a significant decrease (by 40% to 50%) under RCP8.5 compared to baseline. Under RCP2.6, the labor capacity in the 2071–2099 period would be generally similar to that during the 2021–2050 period, showing slightly less labor capacity than the baseline period. The large decreases in labor capacity generally would occur in the regions with high population densities and developed economies.
Martinich and Crimmins (96)	US	Daily maximum temperature	all work sectors for high-risk (agriculture, construction, utilities, and manufacturing) and low-risk labor sectors	RCP4.5 and RCP8.5 in 2050 and 2090 vs. 2003–2007 (baseline)	Lost Labor Hours (millions) Lost wages (USD)	Changes in productivity estimated using the dose–response functions between temperature and working time obtained by literature (91). Number of minutes individuals work from survey data. Losses calculated also due to changes in cold temperature, including extreme temperatures. Economic losses in terms of wages lost calculated by lost labor supply hours, Number of workers adjusted by ICLUSv2 projected population and wages scaled by economic growth.	Lost labor hours under RCP8.5 are 880 (500 to 1, 400) millions in 2050 and 1, 900 (1, 000 to 2, 700) millions in 2090. In economic terms, 44, 000 USD in terms of wages lost in 2050 and 160, 000 USD wages lost in 2090 under RCP8.5. Stronger losses in labor hours and economic losses in Southeast, Midwest, Southern Plains.
Orlov et al. (97)	10 European countries	Wet-bulb Globe Temperature (WBGT)	Outdoor workers (agriculture and construction) for high (400W) and moderate (300W) intensity work	Heat waves August 2003, July 2010, and July 2015 (no climate change scenarios)	Monthly average changes in worker productivity during heat waves (%) Direct economic losses from heat-related reductions in worker productivity (USD 2015 per worker)	Changes in productivity estimated using the dose–response functions between WGBT and working time using the Hothaps exposure-response functions and the ISO standards under. Direct economic losses (or direct private costs) calculated using the sectorial average economy-wide earnings multiplied by relative reductions in worker productivity. Also social costs resulting from heat-related decreases of output were calculated by using a computable general equilibrium (CGE) model	In August of 2003, the mean value of heat-induced reductions in worker productivity in the top fifteen most adversely affected European countries accounted for approximately 3%. In the same month, the mean value of direct economic losses resulting from heat-induced reductions in worker productivity in the agricultural sector in the top ten most affected European countries accounted for approximately 83 USD per worker, whereas in July of 2010, it was 59 USD per worker, and in July of 2015, it was 90 USD per worker. Country specific estimates could be larger, e.g., in the agricultural sector of Italy in 2015 could be large than 1100 USD per worker. With respect to the construction sector, the mean value of direct economic losses in August of 2003 amounted to 61 USD per worker, in July of 2010, it was 41 USD per worker, and in July of 2015, it was 72 USD per worker
Parks and Xu (98) Gray literature	US	Daily mean temperature	Low- and high-risk sectors (farming, fishing and forestry, construction and extraction, Installation Maintenance and Repair, Transportation, and Material Moving Occupations)	1983–2016 (no climate change scenarios)	total cost of lost labor (billions USD and percent of GDP)	Changes in productivity (time lost per day per worker) estimated using the dose–response functions between temperature and working time obtained by literature (91) Number of minutes individuals work from survey data. Labor lost translated into cost by multiplying lost days per worker by the number of workers in each sector and mean wage per minute.	In high-risk sectors, total cost of lost labor from 0.3% in 1983 to 0.58% in 2016 as percentage of total GDP. The total loss of labor productivity in the farming, fishing, and forestry sector was nearly 3 billion USD in 2016. California accounted for 82.60% of the total national loss. In the construction and extraction sector, the total loss amounted up to $34 billion in 2016. Similarly, most of the loss took place in California, Texas and Arizona. Losses in 2016 were 29 billion USD in the installation maintenance and repair occupation and 41 billion USD in the Transportation and Material Moving Occupations sector.
Rao et al. (99)	India	Steadman Heat Index	not specified	2016–2035, 2046–2065, 2080–2099 (RCP4.5 low emission, RCP8.5 high emission) vs. 1986–2005 (baseline) during summer	Decrements in summertime work performance (%)	Labor productivity losses (work hours) estimated using the dose–response functions between temperature and work performance obtained by literature in each grid cell and each region. Projections of future labor productivity losses estimated.	Our assessment showed a decline in work performance to up to 40% under the RCP8.5 and 35% under the RCP 4.5 scenario. The coastal regions of India (east and west coast) are found to be more vulnerable to heat stress impacts by showing a perceptible increase in the heat impact days and a decline of 30 to 40% in the work performance, particularly in east coast region.
Somanathan et al. (100) Gray literature	India	Wet-bulb Globe Temperature (WBGT)	manufacturing industry (weaving, Garment Manufacturing, rail production, diamond polishing, panel of manufacturing industries)	1971–2009 (no climate change scenarios)	Actual efficiency (daily worker output) Absenteeism Annual plant manufacturing output	Empirical estimate of the dose–response functions between WBGT and actual efficiency measured as the actual hourly output averaged over each day, as a measure of the combined productivity of each line of workers based on daily production output and attendance data for workers in local farms. Empirical estimate of the dose–response functions between WBGT and work absenteeism. Empirical estimate of the dose–response functions between WBGT and plant manufacturing Output (by wage share and electricity intensity)	Ambient temperatures have non-linear effects on worker productivity, with declines on hot days of 4 to 9 percent per degree rise in temperature. Sustained heat also increases absenteeism, at the rate of approximately 1 to 2 percent with every additional day of elevated temperatures. Regarding plant output, it declined by between 3 and 6% per degree above 25 °C.
Somanathan et al. (101)	India	Wet-bulb Globe Temperature (WBGT)	manufacturing industry (weaving, Garment Manufacturing, rail production, diamond polishing, Steel mill, panel of manufacturing industries)	1998–2009 (no climate change scenarios)	Change in average worker daily efficiency (%) Absenteeism Annual plant manufacturing output	Empirical estimate of the dose–response functions between WBGT and actual efficiency measured as the actual hourly output averaged over each day, as a measure of the combined productivity of each line of workers based on daily production output and attendance data for workers in local farms. Empirical estimate of the dose–response functions between WBGT and work absenteeism. Empirical estimate of the dose–response functions between WBGT and plant manufacturing Output (by wage share and electricity intensity)	The clearest effects are found for weaving workers, where an additional day above 35°C in the six preceding days causes a 2.7% decrease in contemporaneous daily output and a 0.005 increase in the probability of missing work. The impact of a 1°C increase in temperature on district output was a declines of 3% per 1°C. Declines in daily output on hotter days are seen only in sites without climate control.
Suzuki-Parker et al. (102)	Tokio and Osaka (Japan)	Wet-bulb Globe Temperature (WBGT)	Outdoor light and heavy labor work	2030s, 2050s, 2070s, and 2090s under SRES A1B vs. 2000 (baseline)	Labor hour loss Percent hour losses in climate change scenarios compared to baseline (%)	Labor productivity losses (work hours) estimated using the dose–response functions between temperature and work performance obtained by literature in each grid cell and each region. Projections of future labor productivity losses estimated.	For heavy intensity work, the estimated loss in the hours in the 2070s corresponds to a roughly 60% in Tokyo and 75% in Osaka reduction relative to the 2000s. The reduction rate of labor hours is larger in Osaka than in Tokyo possibly since Osaka there will be a larger temperature increases. The number of heavy labor restricted days (days with minimum daytime WBGT exceeding the safe level threshold for heavy labor) is projected to increase from~5 days in the 2000s to nearly two-thirds of the days in August in the 2090s.
Szewczyk et al. (103)	Europe	Wet-bulb Globe Temperature (WBGT)	4 classes based on occupational vulnerability to heat stress: cognitive and physical work divided in light (200W), moderate (300W), and heavy (400W) labor	2020, 2050, and 2080 for RCP8.5 vs. 1990 (baseline)	Labor productivity change (%) Annual economic losses (euros or proportion of GDP)	Labor productivity losses (work hours) estimated using the dose–response functions between temperature and work performance by occupational category. The regional employment in the four occupational groups, is used to aggregate the occupational losses into a single metric representing regional labor productivity loss by period. A macroeconometric model of the European economy is then used to assess implications of change in productivity in monetary terms. In addition to the direct effect of the labor productivity shock, the model also captures the dynamic, long-term cumulative effects that operate through the capital investment processes. Economic impacts are presented as changes in annual GDP in 2013 Euros. Adaptation was also considered: diffusion of space cooling and increase in the use of robotic exoskeletons.	Productivity of labor can be 1.6% lower in the worst-case scenario (RCP8.5), with the largest reductions in southern European regions. Adaptation can reduce the productivity losses by around 40%, with higher rates of reduction for the lower warming levels. The annual economic losses in Europe could reach 563 billion euros or 1.15% of GDP by the 2080s in the worst-case scenario.
Vivid Economics (104) UK 2017 Gray literature	Ethiopia, Ghana, India, Jordan, Tanzania	Wet-bulb Globe Temperature (WBGT)	Outdoor and indoor: agriculture, manufacturing, construction, other industry, wholesale and retail trade, transport, storage and communication, and other services.	2020 to 2039 and 2040 to 2059 for RCP2.6 (low emission) and RCP8.5 (high emission) vs. 1986–2005 (baseline)	Productivity loss (%) Total employment and ‘equivalent effective workers' lost due to heat stress (percent of GVA)	Labor productivity losses (work hours) estimated using the dose–response functions between WGBT and remaining productivity (%) by different work intensities from Hothaps models and safety standards. Adaptation solutions were also considered (i) a decrease in the supply of labor (total hours worked), (ii) a reduction in the effort applied per hour worked, (iii) a reduction in productivity, per hour worked, for a given level of effort.	These losses are 1–5% of productivity for a 1.5 °C temperature. In all countries except Jordan, the first and second largest absolute reductions in labor productivity loss are in the agriculture and construction sectors, respectively. In India the reduction is 20% of total workforce hours lost due to heat stress, the other countries losses are lower. Changing working hour patterns will be most effective in countries where temperatures are high during ‘normal' working hours, and lower at other times. The split shift reduces productivity losses by between 0.9 and 8 percentage points, equivalent to reductions in lost productivity between 40% and 70% across all five countries.
Xia et al. (105)	Nanjing, China	Humidex	all work sectors (indoor and outdoor)	14-days heat wave 2013 (no climate change scenarios)	Industrial Reduced Productive Working Time (percentage) Economic losses (billion Yuan and proportion of the city Gross Value of Production, GVP)	Interdisciplinary approach by combining meteorological, epidemiological and economic analyses to investigate the macroeconomic impacts of heat waves on the economy. Labor productivity losses (work absences) estimated using the dose–response functions between Humidex and working time loss by different work intensities. Direct losses were inputted in the supply-driven Input-Output model to measure the total indirect economic loss incurred along the production supply chain, which is measured as the total loss in output. Economic loss estimated from monetary value of sector outputs taking into account interdependencies between sectors.	Each heat induced death results in 250 working days lost. Extreme heat also results in a 12% loss of daily working time for indoor workers in the manufacturing and service sectors during the heat wave, while it induces a daily loss of 6 h (45 min times 8 h per day) of working time for outdoor workers in the agricultural, mining, and construction sectors during the heat wave due to the occupational health safety plan. The average percentage reduction in industrial productive working time is 2.50% across all 42 industries in Nanjing in 2013 compared with full productivity and capacity without any heat effect. The greatest losses in industrial productive working time occur in the agricultural (4.50%), mining (4.22%), and construction (4.20%) sectors, where most laborers work outdoors. In economic terms, 27.49 billion Yuan due to the heat wave, 3.43% of Nanjing's GVP in 2013.
Zhang and Shindell (106)	US	Wet-bulb Globe Temperature (WBGT); daily maximum temperature	light, medium, and heavy work	2050 and 2100 (RCP8.5 and RCP4.5) vs. 1980–2016 (baseline)	Economic losses (billions USD in 2016) from reduced labor supply due to extreme heat Economic losses as percentage of GDP (%)	Labor productivity losses (work absences) estimated using the dose–response functions between WBGT (or daily maximum temperature) and working time loss by different work intensities from literature. The calculated work loss was multiplied by the working population in each country to get the annual total labor loss due to heat and by the county-specific hourly wages for each sector.	In the baseline period, on average 421 (95% confidence interval (CI): 70–561) million hours of work were lost annually due to extreme heat across the USA (1.2% of total billion work hours). The average market cost was 14 (2.3–18.7) billion USD. Under the RCP8.5 scenario, 1.5 (0.3–2.1) billion US workforce hours per year will be lost by the end of this century. The market cost associated reach $50 (8.3–66.7) billion per year, more than triple the losses with current climate conditions. The costs increase to 0.18% and 0.30% of the total GDP by the 2050s and the end of the century without accounting for any changes in GDP itself over time. Impacts greater in Southern states.
Zhao et al. (107)	China	High-temperature days: daily maximum temperature exceeding the temperature threshold for high-temperature subsidies to workers (indoor and outdoor)	all work sectors (indoor and outdoor)	2030, 2040, 2090 (RCP2.6, RCP4.5, RCP 8.5) vs. 1979–2005 (baseline)	Labor losses (billions Yuan) Losses as percentage of GDP (%)	High-temperature subsidies (HTSs) are estimated based on the formal employee to total population ratio, the daily subsidy at the jth class in yuan per person per day, the frequency of HTDs at the jth class and ith grid point in days per year. The daily HTS are then summed up per year over grid points. The HTS values in yuan per employee per year are calculated from annual HTS per grid cell, divided by the national total number of formal employees.	On average, the total HTS in China is estimated at 38.6 billion yuan/y (6.22 billion USD per year) over the 1979–2005 period, which is equivalent to 0.2% of the gross domestic product (GDP). Assuming that the HTS standards (per employee per hot day) remain unchanged throughout the 21st century, the total HTS may reach 250 billion yuan/y in the 2030s and 1, 000 billion yuan/y in 2100. Without specific adaptation, the increased HTS cost is mainly determined by population growth until the 2030s and climate change increase in hot weather.
Zivin and Neidell (91)	US	daily mean temperature	Outdoor and indoor high- and low-risk sectors. High-risk industries: agriculture, forestry, fishing, and hunting, mining, construction, manufacturing, and transportation and utilities industries; Low risk: remaining industries	2003–2006 (no climate change scenario evaluated)	Time allocated to labor market activities or leisure activities (min)	Econometric model of percent of time allocated to labor market activities and percent of time allocated to outdoor/indoor leisure activities as a function of temperature. Adaptation contribution (e.g., shifting activities across days) was also evaluated for outdoor work.	In high-risk industries, for labor supply, there is little response to temperatures below 80 degrees, but monotonic declines in labor supply above 85 degrees. At temperatures over 100 degrees, labor supply drops by a statistically significant 59 min as compared to 76–80 degrees. At high temperatures, workers appear to substitute their labor supply for indoor leisure, with surprisingly no decline in outdoor leisure. For low-risk industries while there is a decrease in labor supply at temperatures above 95 degrees, this effect is modest and not statistically significant. Little or no role for adaptation measure (intertemporal substitution in the workplace) to mitigate the decrease in labor supply in high-risk industries.

PPP, GDP per capita, purchasing power parity (PPP) per capita.; CGE, computable general equilibrium model; USD, US dollars; RCP, Representative Pathway Concentration Scenarios.; SSP, Shared Socioeconomic Pathways Scenarios.; BaU, Business-as-Usual Scenario.; SRES, IPCC Special Report Emission Scenarios.; GDP, gross domestic product.; GVP, gross value of production.; GVA, gross value added.; GRP, gross regional product.

#### 3.3.1. Global studies

Studies evaluating global economic impacts of current and future occupational heat (*n* = 21) were both scientific publications (59, 60, 63–65, 67–70, 72, 74–80) and gray literature (16, 61, 62, 66), providing evidence of heat-related reductions in work productivity at the global level. Productivity losses associated with climate change by 2100 under the worst-case scenario (high emissions) range from nearly 10% (68) to 30–40% (15, 65, 67, 97) at the global level. GDP losses for the same period and scenario varied between 1.8% compared to baseline (75) to 23% (59). In specific sectors such as agriculture, the loss of productivity expressed as a percentage reduction in GDP is even >30–50% (64, 74). Global studies provided also estimates for the different world regions, by highlighting higher impacts from both current and future climates in low- and middle-income countries (60–62, 67, 68, 78), like sub-Saharan Africa (63, 64, 80), very hot countries (59, 66), and high-intensity work in low-latitude countries (75).

#### 3.3.2. Regional studies

Regional studies (*n* = 28) were also considered both from peer-reviewed journals (73, 81, 82, 87–89, 91, 93–97, 99, 101–103, 105–107) and the gray literature (83–86, 90, 92, 98, 100, 104) confirming a heterogeneous impact of heat on work productivity not only among countries but also within the same country (82, 95, 99, 102). As seen in the global studies, low-latitude, high-intensity labor settings were the most affected such as West Africa, Southeast Asia, and Central and South America. Moreover, specific local studies suggest an impact also in other regions such as southern European countries (92, 97, 103), some parts of the US (especially agricultural areas in Southeast and Southwest) (94, 96, 106), and Australia (86). For example, under medium-high emission scenario by the end of the century, a 0.4–0.9% loss in productive days was shown for Southern Europe (92), 10.2% of wages lost were estimated in the US (94), and a 16–17% labor capacity loss was predicted in China (95). Agriculture was the sector most affected by heat stress, both considering the current climate and future scenarios and among non-agricultural sectors, construction, manufacturing, transportation, service, and mining (73, 83, 86, 98, 104, 105). Agriculture (97) and manufacturing sector are also expected to be impacted in terms of farm production output losses (100, 101).

The evaluation of adaptation measures was marginally evaluated: Air conditioning was effective in reducing labor productivity losses in indoor settings in two European studies (84, 103), with one study suggesting also a potential role for technological measures such as robotic exoskeletons (103), while measures affecting the work/rest schedule have been shown to reduce productivity loss in outdoor workers in one study in Ethiopia, Ghana, India, Jordan, and Tanzania (104) and in one US study (94) while another US study provided uncertain results (91).

## 4. Discussion

This literature review provides an updated summary of the evidence on socioeconomic impacts of occupational heat exposure and confirms the results of previous reviews (7, 11, 12, 14–16) and of the latest IPCC report (8). The review also provides further evidence on the association between indoor and outdoor heat exposure and socioeconomic impacts in terms of productivity loss or costs. Throughout the different study types, a coherent picture of the social and economic impacts of heat exposure in the workplace emerges, highlighting the main pathways for heat-related productivity losses. One pathway is in common with the general population and is related to the increased risk of acute heat-related illnesses and deaths (1) and the emergence of chronic illnesses consequences such as renal impairment (5, 6). Underlying biological mechanisms include thermoregulatory failure with cardiovascular fatigue and respiratory distress, dehydration with progressive kidney dysfunction in case of sustained chronic exposure. Another pathway is related to changes in vigilance and cognitive performance that may enhance the risk of distraction, impairment in risk perception, and reaction time leading to improper operation and injury (3). The third pathway directly related to work productivity and physical performance reductions and to the physiological need to rest during heat exposure, leading to a reduction in work hours and work output (16). All these pathways are strongly interconnected, and it is difficult to identify which plays a major role in productivity loss.

The most robust evidence in the present review derives from time-series or case-crossover studies (53, 54, 58). Such methods are the “gold-standard” study design to evaluate the short-term effects of environmental exposures at the population level while controlling for time-varying confounders. Field studies represent an important piece of evidence about heat-related productivity loss, but they have the limitation of providing evidence on a small sample and related to given setting at a specific time interval (110) and only a limited number of studies adjust for potential confounders (22, 39, 40, 47). Studies are consistent in reporting labor productivity loss perceived by the workers (19, 20, 23, 29, 32–35, 43, 45, 46, 48, 49) and have negative impacts in terms of physical performance (42) and work output (21, 25).

The largest body of evidence from the present review comes from economic modeling studies. These are mostly global or regional studies which apply modeled spatially resolved temperature data for the current or future climate change scenarios to risk functions from physiological studies (65, 71, 89, 91, 108) to obtain an estimation of loss in working hours which is then converted to economic costs *via* workers' wages or as portion of gross domestic product (GDP). Studies combining economic and climate modeling have the added value of providing current and future impact estimates which are useful for the definition of adaptation and mitigation actions. However, these models are dependent on the scenarios selected and assumptions made; thus, it is important that the uncertainty is adequately reported (71). More complex economic models, i.e., the general equilibrium models (109), are able to account for the interdependencies among sectors but also have a number of methodological challenges in particular in accounting for societal welfare changes (different by GDP), non-linear damages, and micro- and macro-adaptation processes (8). Although methodological differences limit comparability, actual productivity losses at the global level are nearly 10% (62, 65) and under the worst-case scenario (high emissions) by 2100 are expected to increase up to 30–40% (62, 65, 67, 75). GDP losses for the same period and scenario varied between 1.8% compared to baseline (75) and 23% (59). Scenarios suggest that in regions like sub-Saharan Africa, India, Southeast Asia, and South America, productivity losses may be even greater as they will experience significant warming and a high share of the economy entails labor-intensive occupations (59–64, 66–68, 75, 78, 80), experiencing over a 10 times increase in work hours lost under the worst emission scenario (62). Some studies also report substantial reductions in work capacity in the United States, Europe, and Australia (86, 92, 94, 96, 97, 103, 106).

Vulnerability factors increasing the risk of heat-related productivity loss may differ according to the underlying causal pathway, with potential differences among factors increasing vulnerability for heat-related diseases, heat-related injuries, and heat-related productivity loss. However, the link with socioeconomic impacts is less clear. Individual factors such as age (53, 56), gender (31, 37, 45, 49), race (52), education level (37, 53), immigration status (34), and comorbidities such as kidney failure or other conditions (21, 22) have been related to higher reduction in work productivity in some studies, but the evidence is limited. The work environment may also affect worker susceptibility to productivity losses related to heat, as consistently shown in the literature. Some occupational sectors, primarily agriculture and construction, appear more affected than others, suggesting a higher impact on productivity loss due to more intense physical activities. The agricultural sector alone accounts for two-thirds of all labor hours lost globally in 2021 at the global level (78). Other sectors or workers affected include transportation and utilities (83, 98), miners (37, 58, 83, 86, 105), and indoor workers with no air conditioning (19, 100, 101). Furthermore, performing heavy tasks (45, 48, 68, 70, 75, 89, 93), direct sunlight exposure (36, 63, 89), and use of personal protective equipment (PPE) (23, 29, 35) have been associated with productivity loss. In some cases, the work sector and task may be a multiplier of existing individual vulnerabilities, as in the case of migrant agricultural workers (34) or young manual workers (51).

Awareness of heat-related risks, health and safety actions and training, as well as workers behaviors play a key role productivity loss due to heat among workers (37). The heterogeneous perception of heat-related occupational risks and causes of productivity loss (35, 37, 45, 46) suggests that more efforts are needed to enhance risk perception and heat-protective behaviors. Work management policies need to have a holistic approach by addressing all potential pathways linking heat exposure to workers' health, safety, and productivity (37). Specific information tools aimed to increase adaptive capacity and protective behavior especially in the most vulnerable workers can reduce impacts on productivity, as suggested by the work carried out in Italy within the Worklimate project (https://www.worklimate.it/en/home-english/).

Some strengths and limitations are worth mentioning: the quality of studies was not formally evaluated, and the search was restricted to only two bibliographic databases (PubMed and Web of Science) and only to English language studies that may have restricted the geographical coverage of some areas of the world such as Central and South America and Africa. To partially counterbalance this, the inclusion of a significant number of studies (14 out of 89) from the gray literature (from academia, NGOs, or economic or policy organizations) (16, 46, 61, 62, 66, 83–86, 90, 92, 98, 100, 104) retrieved from reviews in the field (7, 11, 12, 14–16) ensures to include a greater number of studies from low- and middle-income countries where the issue is particularly relevant. Moreover, the scoping review was limited to studied published since 2010, but this was also the publication horizon from previous reviews (11, 12, 14).

Due to the heterogeneity of studies in terms of methodologies used, heat exposure indicators, and economic cost measures, a quantitative synthesis was not possible. However, the present literature review provides a clear and consistent indication of the effects of heat on productivity and costs for employers and employees, economic sectors, social security systems, and national economies. The impacts are coherent across a range of study designs and study areas although we cannot exclude that some relevant papers are missing, the possibility that publication bias could distort these results is low thanks to the inclusion of a relevant piece of gray literature as specified above. This large body of evidence can support decision-making process in terms of improving and protecting worker safety, health, and wellbeing following the Total Worker Health approach (111) also in the context of climate change resilience and response by involving all relevant stakeholders both at the policy level and at the workplace level (i.e., nurses or other healthcare practitioners and workers' compensation professionals) (112). A number of initiatives in this field have been taken, but more efforts are needed in terms of prevention, employer and employee information, and training to raise awareness and increase resilience and behavioral adaptation. The evidence suggests that the expected impacts of climate change may be even greater and that investing resources in prevention actions in occupational settings has both social and economic benefits. Despite the consistent evidence on productivity impacts, some knowledge gaps emerge. Future research needs to address them such as the role of individual and work-related factors in increasing worker's vulnerability to productivity losses, and the evaluation of adaptation measures such as work schedule adjustments and work-level reductions only little evaluated in terms of productivity improvements (91, 94, 104).

## 5. Conclusion

In conclusion, much knowledge has been accumulated about heat-related reduction in work capacity in recent years. There is an urgent need for holistic work management policies such as the Total Worker Health approach and for climate change adaptation and mitigation efforts to protect workers' health from future warming and climate extremes, especially in most vulnerable agriculture, manufacturing, and construction sectors and in very hot countries with high-intensity work.

## Author contributions

MDS: literature review, conceptualization, and paper writing. Fd'D: literature review, conceptualization, design, supervision, interpretation, and paper writing. MB, ML, and MM: interpretation and paper writing. AM and FA: paper writing. PM: conceptualization, supervision, and interpretation. All authors contributed to the article and approved the submitted version.
