# Supplementary material for: Occupational heat stress, heat-related effects and the related social and economic loss: a scoping literature review
Source: Front Public Health. 2023 Aug 2;11:1173553. doi: 10.3389/fpubh.2023.1173553 (PMC10434255; doi:10.3389/fpubh.2023.1173553)
Supplement: Supplementary file 1 [file Table_1.DOCX]

**Supplementary table 1. Search strategy**

| **Database: Pubmed** - Search launched in April 2022. | |
| --- | --- |
| **Exposure (heat, high temperature, heatwave, climate change)** | |
| #1 | "Hot Temperature"[Mesh] |
| #2 | (Heat[Title/Abstract] AND (exposure[Title/Abstract] OR stress[Title/Abstract] OR strain[Title/Abstract])) |
| #3 | hot[Title/Abstract] AND weather[Title/Abstract] |
| #4 | (hot[Title/Abstract] OR summer[Title/Abstract] OR high[Title/Abstract] OR extreme[Title/Abstract] OR ambient[Title/Abstract]) AND temperature*[Title/Abstract] |
| #5 | heatwave*[Title/Abstract] OR WBGT[Title/Abstract] |
| #6 | heat[Title/Abstract] AND wave*[Title/Abstract] |
| #7 | climat*[Title/Abstract] AND (change*[Title/Abstract] or variat*[Title/Abstract]) |
| #8 | #1 or #2 or #3 or #4 or #5 or #6 |
| **Population (workers)** | |
| #9 | Work*[Title/Abstract] OR employ*[Title/Abstract] OR labour*[Title/Abstract] OR labor*[Title/Abstract] or occupation*[Title/Abstract] or job*[Title/Abstract] |
| #10 | "Occupational Groups"[Mesh] |
| #11 | #9 OR #10 |
| **Outcomes (costs, productivity, social impacts)** | |
| #12 | "Occupational Injuries/economics"[Mesh] |
| #13 | "Cost of Illness"[Mesh] |
| #14 | (impact*[Title/Abstract] OR burden[Title/Abstract] OR toll[Title/Abstract] OR benefit[Title/Abstract] OR gain*[Title/Abstract]) AND (Socio*[Title/Abstract] OR social*[Title/Abstract] societ*[Title/Abstract] OR economic*[Title/Abstract] OR economy[Title/Abstract]) |
| #15 | cost*[Title/Abstract] |
| #16 | (sick*[Title/Abstract] OR disability[Title/Abstract] OR injury[Title/Abstract] OR accident[Title/Abstract]) AND (leave*[Title/Abstract] OR allowance[Title/Abstract] OR compensation[Title/Abstract]) |
| #17 | productiv*[Title/Abstract] OR efficiency[Title/Abstract] OR absenc*[Title/Abstract] OR absent*[Title/Abstract] OR loss*[Title/Abstract] |
| #18 | "Absenteeism"[Mesh] |
| #19 | "Efficiency"[Mesh] |
| #20 | #12 or #13 or #14 or #15 or #16 or #17 or #18 or #19 |
| #21 | #8 AND #11 AND #20 |
| #22 | #21 AND "Humans"[Mesh] |

| **Database: Web of science** - Search launched in April 2022. | |
| --- | --- |
| **Exposure (heat, high temperature, heatwave, climate change)** | |
| #1 | TI=(heat AND (exposure OR stress OR strain)) |
| #2 | AB=(heat AND (exposure OR stress OR strain)) |
| #3 | TI=(hot AND weather) |
| #4 | AB=(hot AND weather) |
| #5 | TI=((hot OR summer OR high OR extreme OR ambient) AND temperature*) |
| #6 | AB=((hot OR summer OR high OR extreme OR ambient) AND temperature*) |
| #7 | TI=(heatwave* or wbgt) |
| #8 | AB=(heatwave* or wbgt) |
| #9 | TI=(heat AND wave*) |
| #10 | AB=(heat AND wave*) |
| #11 | TI=(climat* AND (change* or variat)) |
| #12 | AB=climat* AND (change* or variat)) |
| #13 | #1 or #2 or #3 or #4 or #5 or #6 or #7 or #8 or #9 or #10 or #11 or #12 |
| **Population (workers)** | |
| #14 | TI=(Work* OR employ* OR labour* OR labor* or occupation* job*) |
| #15 | AB=(Work* OR employ* OR labour* OR labor* or occupation* job*) |
| #16 | #14 OR #15 |
| **Outcomes (costs, productivity, social impacts)** | |
|  | TI=((impact* OR burden OR toll OR benefit OR gain*) AND (Socio* OR social* OR societ* OR economic* OR economy)) |
|  | AB=((impact* OR burden OR toll OR benefit OR gain* ) AND (Socio* OR social* OR societ* OR economic* OR economy) ) |
|  | TI=( cost*) |
|  | AB=( cost*) |
|  | TI= ((sick* OR disability OR injury OR accident) AND (leave* OR allowance OR compensation) ) |
|  | AB= ((sick* OR disability OR injury OR accident) AND (leave* OR allowance OR compensation) ) |
|  | TI=(productiv* OR efficiency or absenc* OR absent* OR loss*) |
|  | AB=(productiv* OR efficiency or absenc* OR absent* OR loss*) |
|  | #12 or #13 or #14 or #15 or #16 or #17 or #18 or #19 |
|  | #8 AND #11 AND #20 |
