# Supplementary material for: Occupational heat stress, heat-related effects and the related social and economic loss: a scoping literature review
Source: Front Public Health. 2023 Aug 2;11:1173553. doi: 10.3389/fpubh.2023.1173553 (PMC10434255; doi:10.3389/fpubh.2023.1173553)
Supplement: Supplementary file 2 [file Data_Sheet_1.docx]

**Supplementary Figure 1. PRISMA Flow diagram of studies selection.**

Records excluded
(n = 8014)

Records screened
(n = 8151)

Records after duplicates removed
(n =8151)

Source: Page MJ, McKenzie JE, Bossuyt PM, et al. The PRISMA 2020 statement: an updated guideline for reporting systematic reviews. BMJ 2021;372:n71.

Additional records identified through other sources
(n = 104)

Records identified through Pubmed and WoS searching
(n = 8431)

Full-text articles excluded, with reasons
(n =48)

Full-text articles assessed for eligibility
(n = 137)

Studies included in qualitative synthesis
(n = 89)

Included

Eligibility

Screening

Identification
